# Supplementary figures and images for: Resource selection of a nomadic ungulate in a dynamic landscape
Source: PLoS One. 2021 Feb 12;16(2):e0246809. doi: 10.1371/journal.pone.0246809 (PMC7880454; doi:10.1371/journal.pone.0246809)

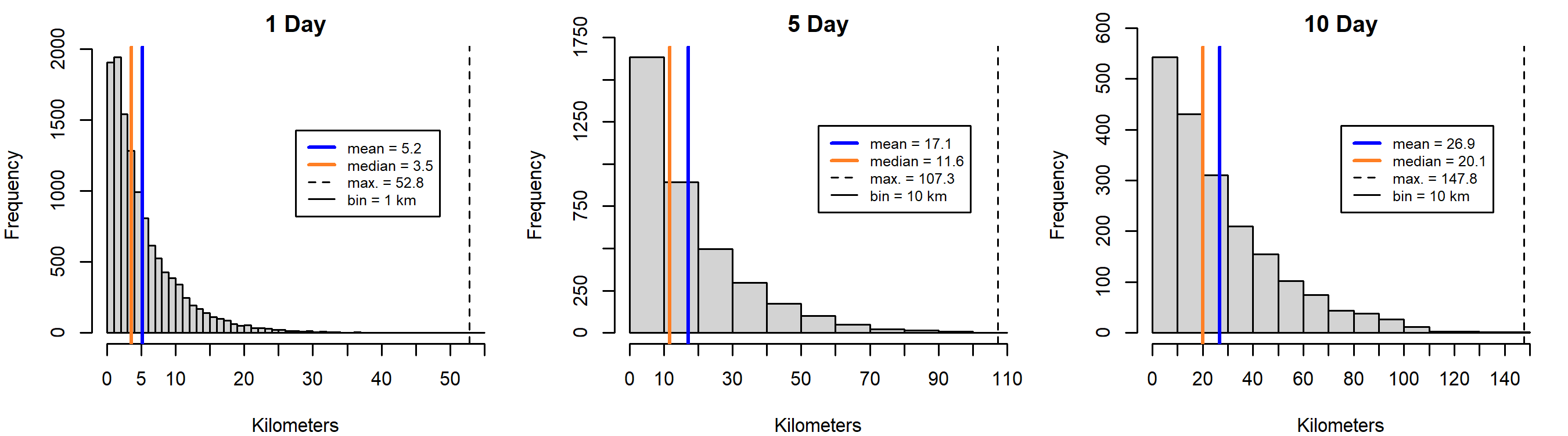

Supplement: S1 Fig — Displacement is the straight line distance between two GPS points. It does not account for the distance the individual actually traveled to move between those two points. (TIFF) [file pone.0246809.s001.tiff]

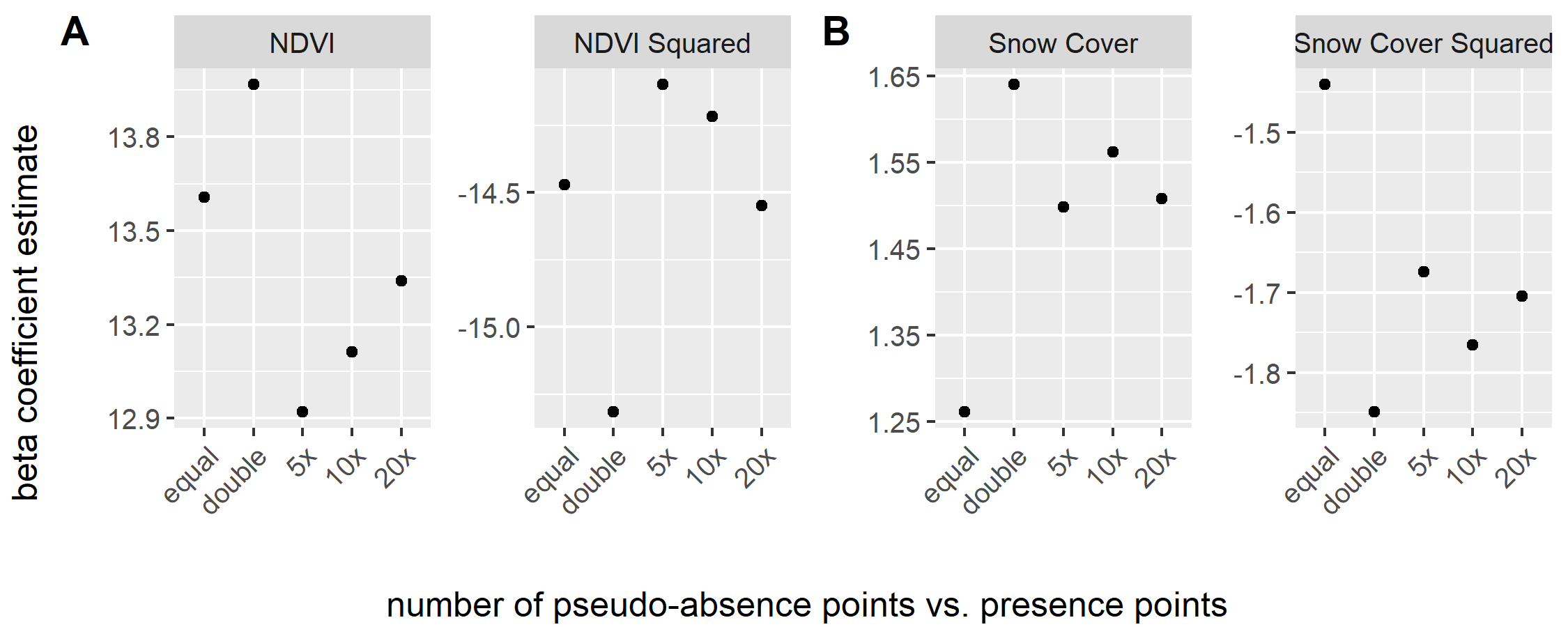

Supplement: S2 Fig — Models were run with an equal number of presence and pseudo-absence points, double the number of pseudo-absence points as presence points, and 5, 10, and 20 times the number of pseudo-absence points as presence points. This was done for all models. Here we show the results for the RSF for each season which had the most support based on AIC. A) For the growing season this was a quadratic model (NDVI + NDVI2) with a random effect for NDVI scene. B) For winter, this as a quadratic model (snow cover + snow cover2) with a random slope and intercept for individual gazelles and a random intercept for year. Beta coefficients for the linear and quadratic parameter are shown. Differences between 10 and 20 times the number of pseudo-absence points are small so we present results of models run with 20 times more pseudo-absence points than presence points. (TIFF) [file pone.0246809.s002.tiff]

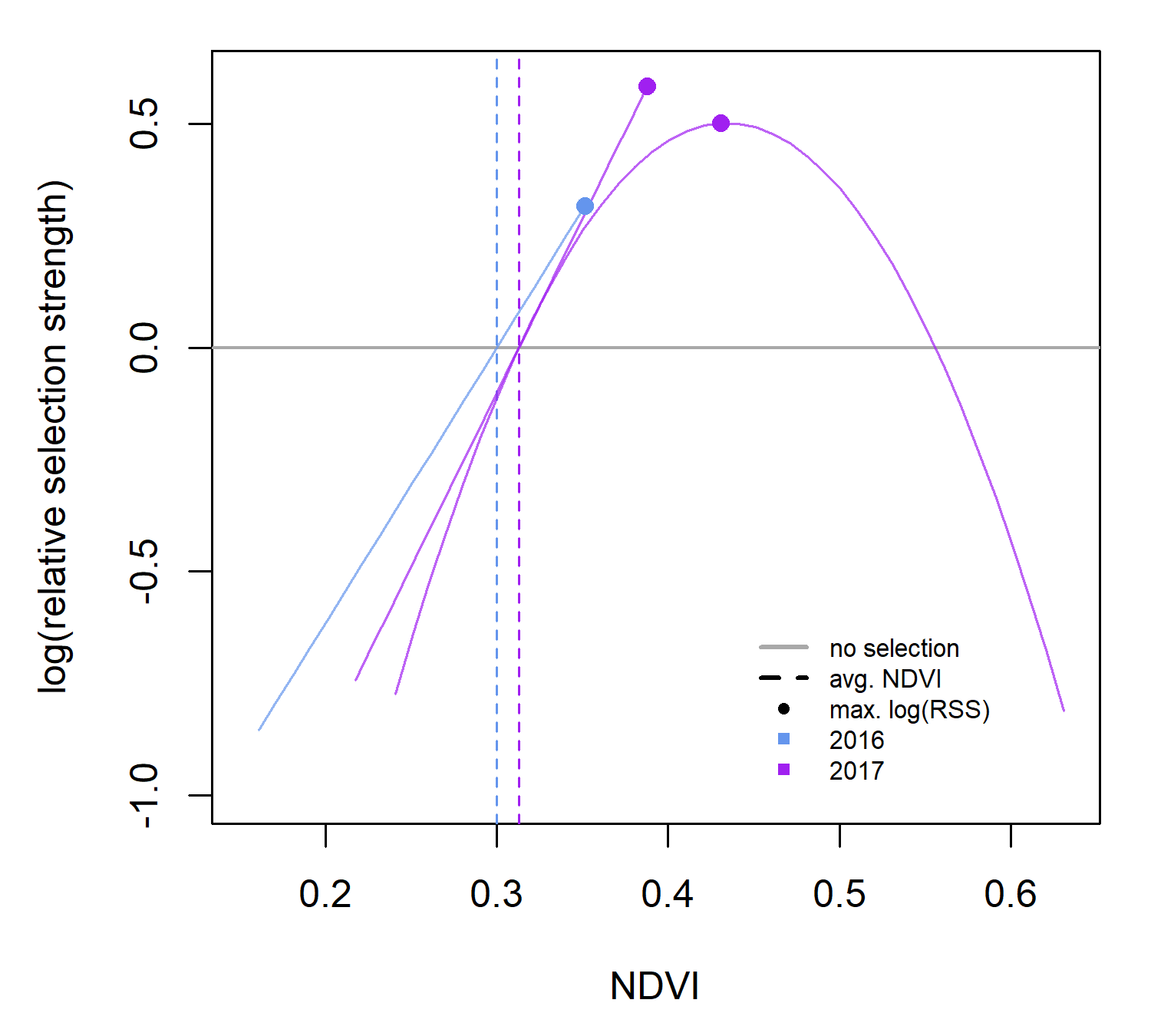

Supplement: S3 Fig — Selection is quantified as relative selection strength (RSS). Curves were plotted based on the results of the individual-year SSFs and show the best fit model for a given individual-year combination. Only those individuals who showed significant selection at the 1 day step-scale are shown. The vertical lines indicate the mean NDVI for a given year (as described in the methods) and were used as the reference points to create the curves. Dots indicate the NDVI values at which RSS peaks. The curve is only plotted for the range of NDVI available to the individual gazelle as defined in the methods. Positive log(RSS) values indicate selection for that NDVI value over the mean NDVI value, negative log(RSS) values indicate avoidance of that NDVI value when compared to the mean NDVI value. (TIFF) [file pone.0246809.s003.tiff]

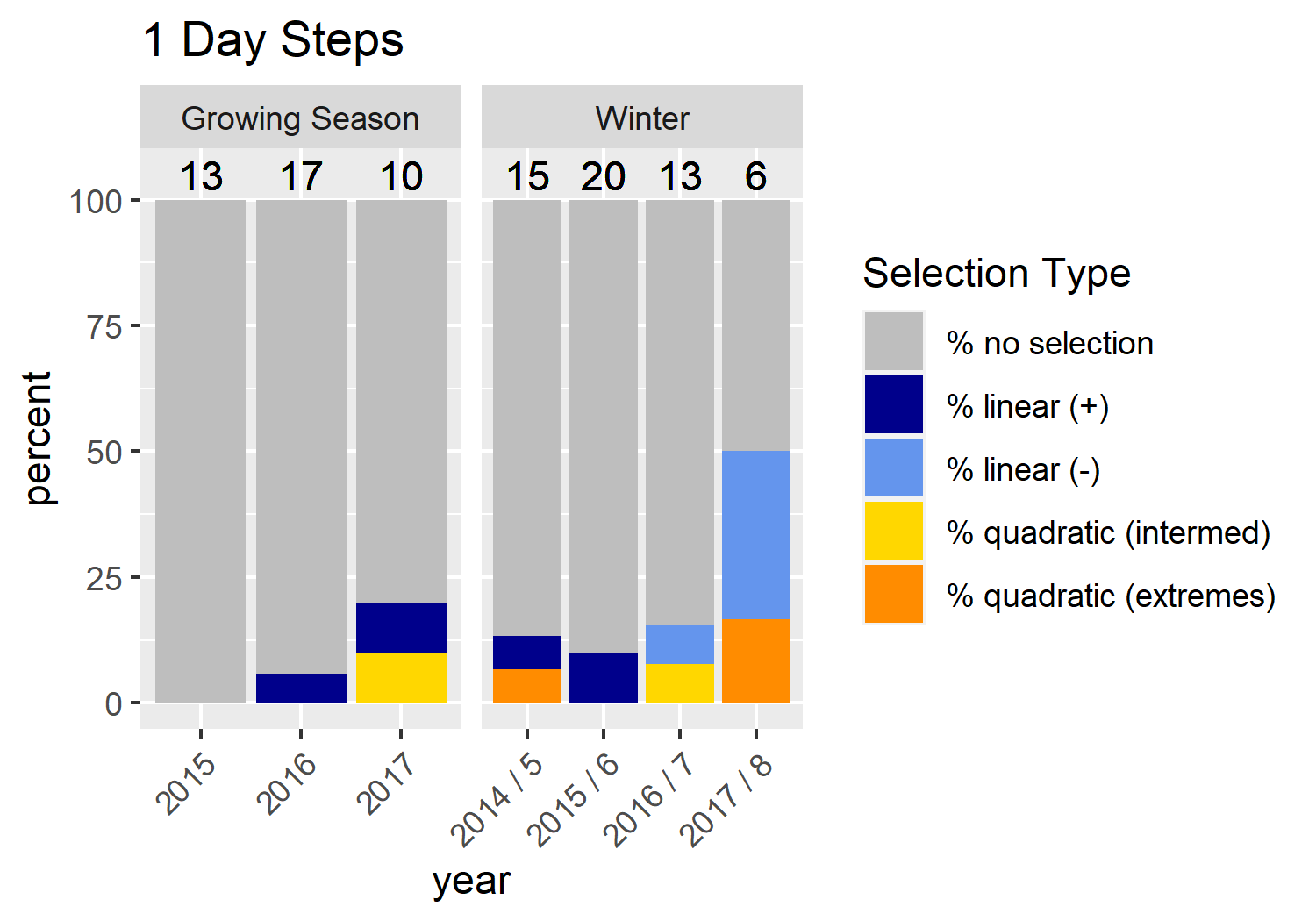

Supplement: S4 Fig — The majority of Mongolian gazelles did not show selection at the 1 day step scale. Blue, yellow, and orange sections of the bar plots show the percent of individuals selecting for NDVI during the growing season of a given year or for snow cover during winter of a given year, grey sections show the percent of individuals that did not show selection. For those individuals showing selection, dark blue indicates selection for the highest NDVI (or snow cover) available, light blue indicates selection for the lowest NDVI (or snow cover) available, yellow selection for intermediate values, and orange selection for high and low values. Above each bar are the number of individuals which had sufficient data (minimum of 20 steps) to run step-selection functions on an individual-year basis. (TIFF) [file pone.0246809.s004.tiff]

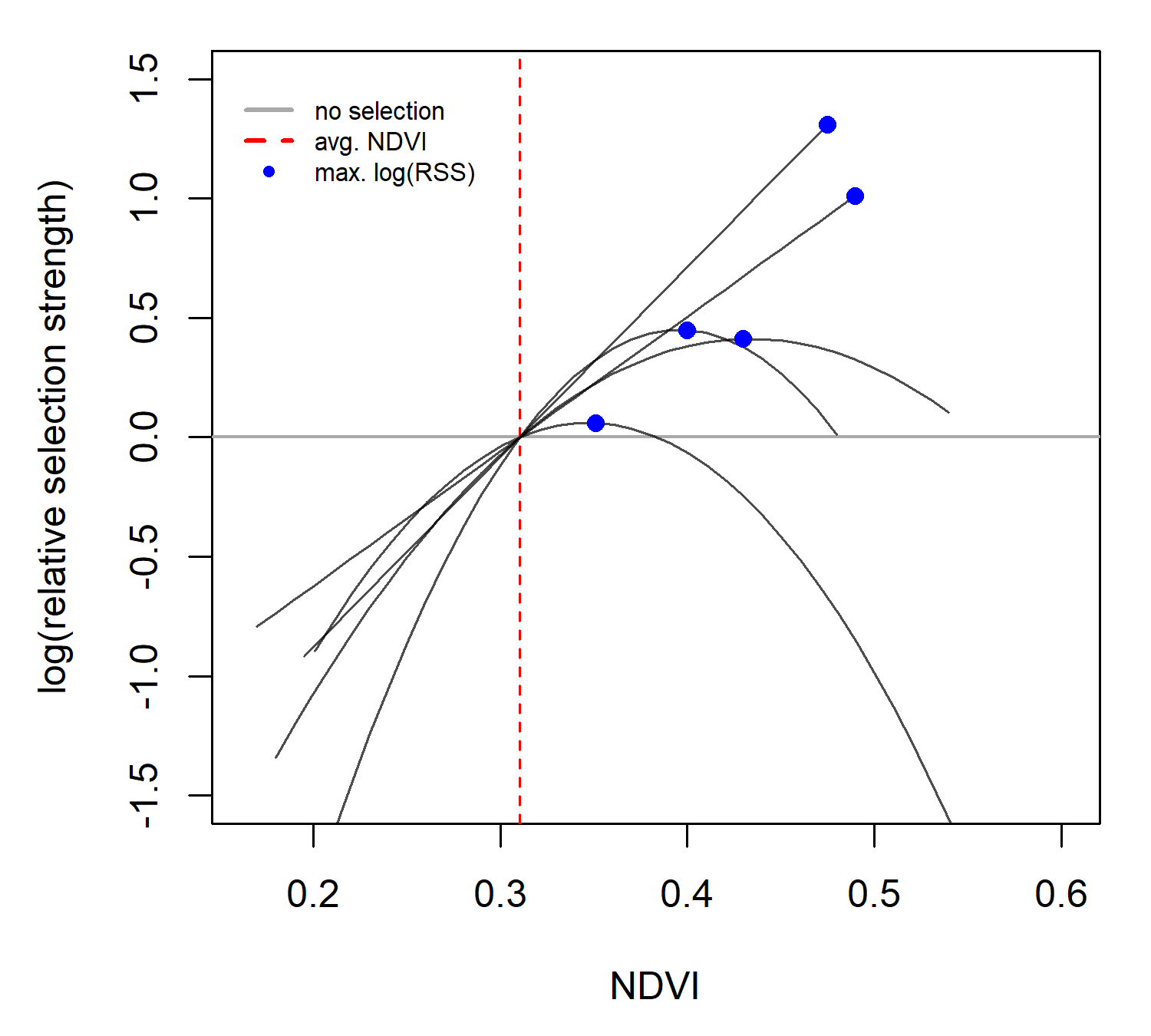

Supplement: S5 Fig — Selection for NDVI at the 5 day step scale shown by individual Mongolian gazelles during the growing season (May 9 –Aug. 29). Selection is quantified as relative selection strength (RSS). Curves were plotted based on the results of the individual SSFs and show the best fit model for an individual. Only those individuals who showed significant selection at the 5 day step-scale are shown. The vertical line indicates the mean NDVI (as described in the methods), which was used as the reference point to create the curves. Dots indicate the NDVI values at which RSS peaks. The curve is only plotted for the range of NDVI available to the individual gazelle as defined in the methods. Positive log(RSS) values indicate selection for that NDVI value over the mean NDVI value, negative log(RSS) values indicate avoidance of that NDVI value when compared to the mean NDVI value. (TIFF) [file pone.0246809.s005.tiff]

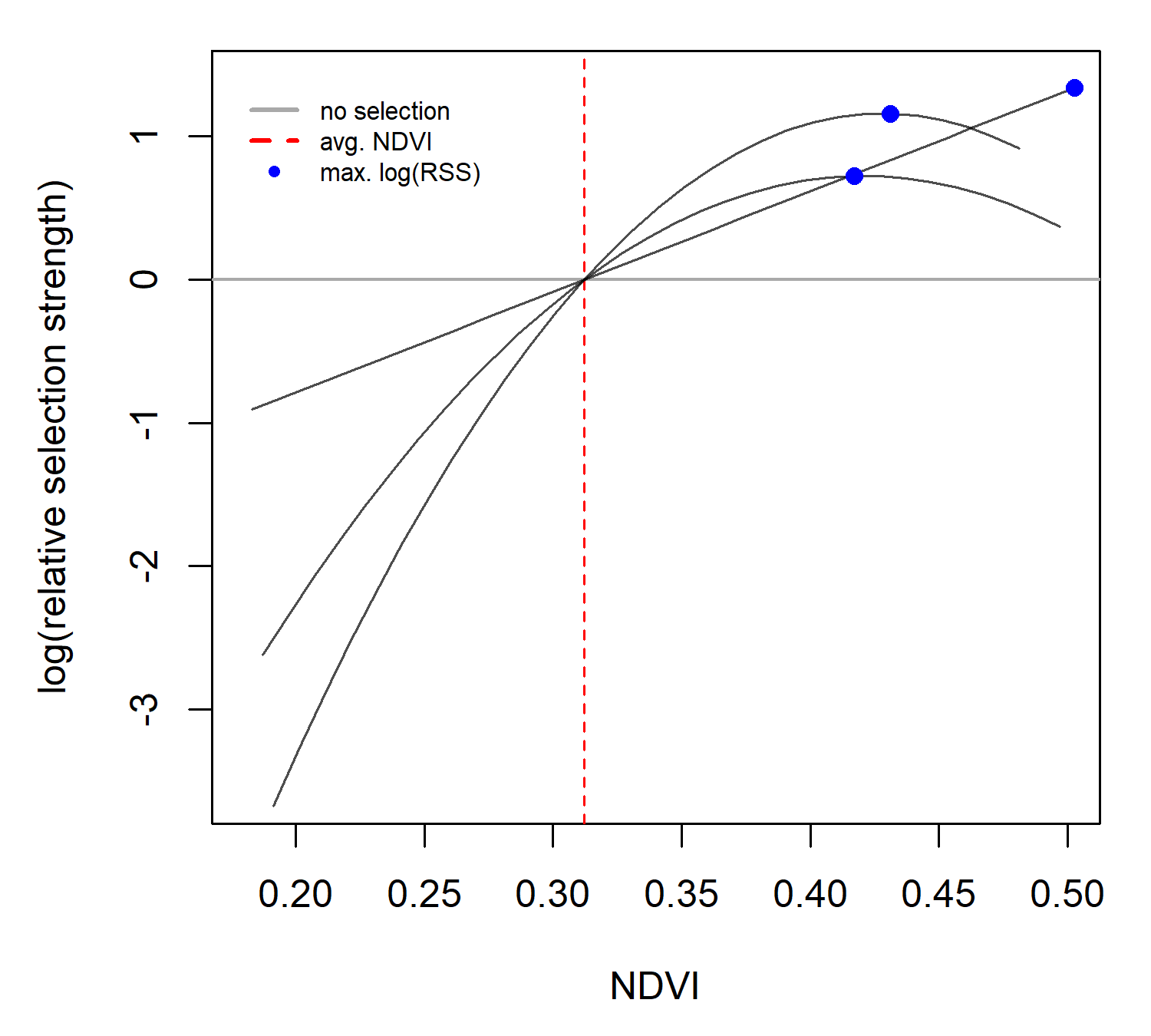

Supplement: S6 Fig — Selection is quantified as relative selection strength (RSS). Curves were plotted based on the results of the individual SSFs and show the best fit model for an individual. Only those individuals who showed significant selection at the 10 day step-scale are shown. The vertical line indicates the mean NDVI (as described in the methods), which was used as the reference point to create the curves. Dots indicate the NDVI values at which RSS peaks. The curve is only plotted for the range of NDVI available to the individual gazelle as defined in the methods. Positive log(RSS) values indicate selection for that NDVI value over the mean NDVI value, negative log(RSS) values indicate avoidance of that NDVI value when compared to the mean NDVI value. (TIFF) [file pone.0246809.s006.tiff]

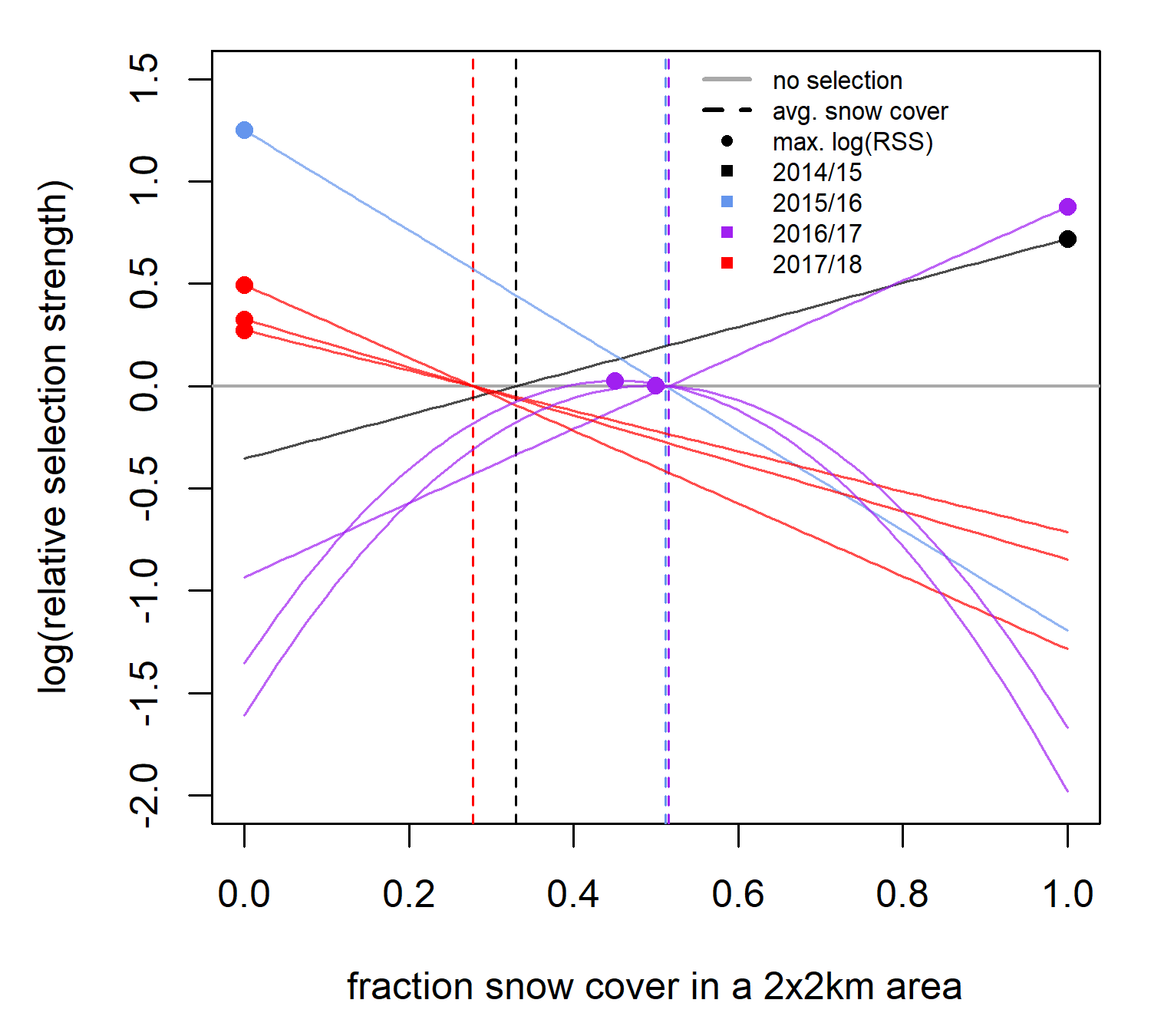

Supplement: S7 Fig — Selection was quantified as relative selection strength (RSS). Curves were plotted based on the results of the individual SSFs and show the best fit model for an individual. Only those individuals who showed significant selection at the 5 day step-scale are shown. The vertical lines indicate mean snow cover in a given year (as described in the methods) and were used as the reference point to create the curves. Dots indicate the snow cover values at which RSS peaks. The curve is only plotted for the range of snow cover available to the individual gazelle as defined in the methods. Positive log(RSS) values indicate selection for that snow cover value over the mean snow cover value, negative log(RSS) values indicate avoidance of that snow cover value when compared to the mean snow cover value. (TIFF) [file pone.0246809.s007.tiff]

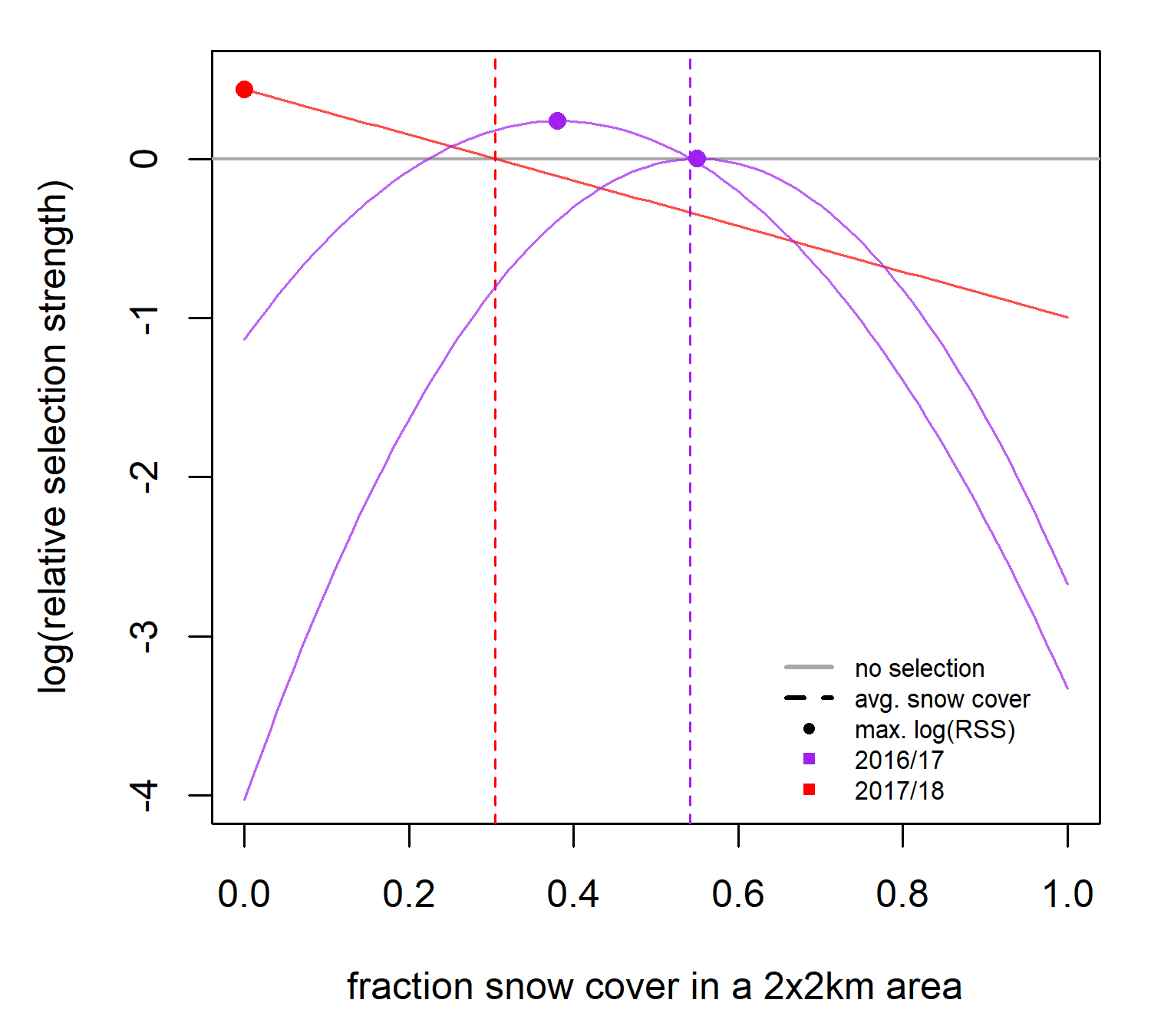

Supplement: S8 Fig — Selection was quantified as relative selection strength (RSS). Curves were plotted based on the results of the individual SSFs and show the best fit model for an individual. Only those individuals who showed significant selection at the 10 day step-scale are shown. The vertical line indicates mean snow cover (as described in the methods), which was used as the reference point to create the curves. Dots indicate the snow cover values at which RSS peaks. The curve is only plotted for the range of snow cover available to the individual gazelle as defined in the methods. Positive log(RSS) values indicate selection for that NDVI value compared to the mean, negative log(RSS) values indicate avoidance of that NDVI valued compared to the mean. (TIFF) [file pone.0246809.s008.tiff]
