# Supplementary material for: Resource selection of a nomadic ungulate in a dynamic landscape
Source: PLoS One. 2021 Feb 12;16(2):e0246809. doi: 10.1371/journal.pone.0246809 (PMC7880454; doi:10.1371/journal.pone.0246809)
Supplement: S1 Appendix — (PDF) [file pone.0246809.s018.pdf]

# S1 Appendix: Effect of Number of Random Points on Step-Selection Function Coefficients

SSFs were run with 25, 50, 100, 200, and 400 pseudo-absence points for each step. A linear shape tests whether individual gazelles select for low or high NDVI within a given year. A quadratic model tests whether individual gazelles select for intermediate NDVI within a given year. Graphs show the model coefficients for each parameter ± their standard error and the p-values. Here we add the standard errors calculated by `clogit()` to the beta coefficients but it should be noted that they must be interpreted with caution because their calculation assumes that the steps are independent, which they are not.

## Growing Season Results

Linear model: presence as a function of NDVI
Quadratic model: presence as a function of NDVI + $NDVI^2$

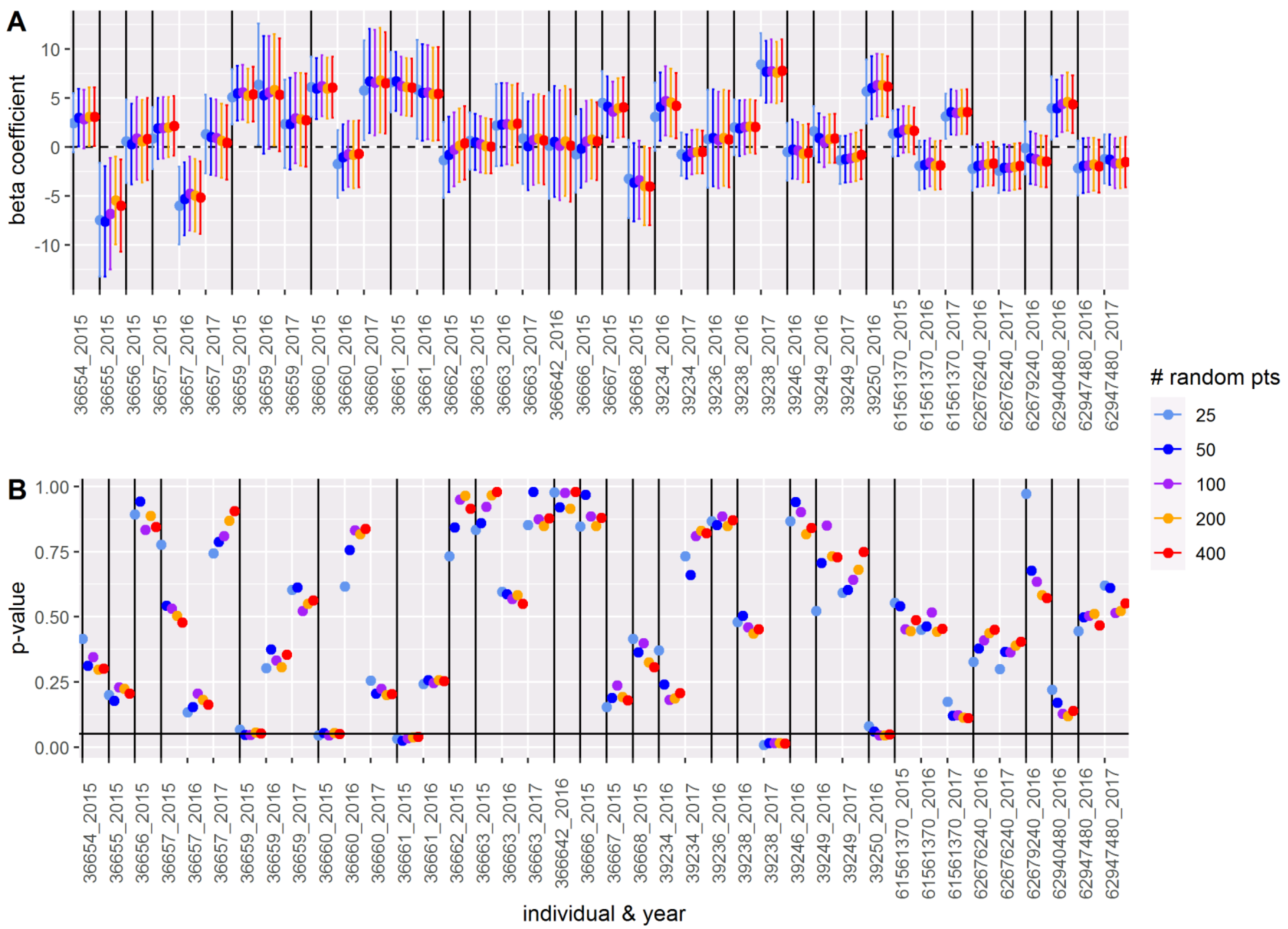


**Figure 1. Results (A - beta coefficients, B - p-values) of SSF run by individual/year at the 1 day scale, testing if selection takes a linear shape in the growing season.**

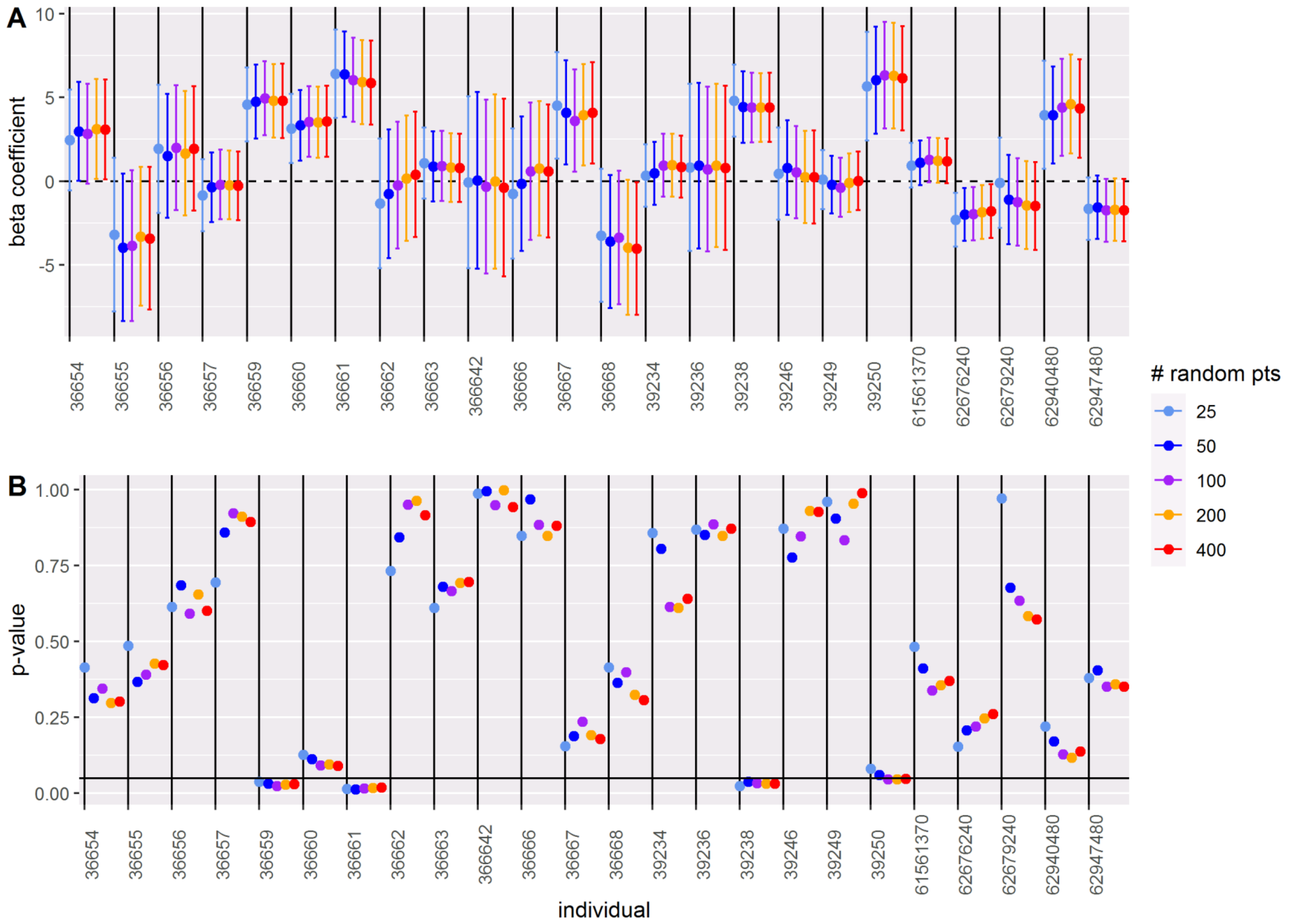


**Figure 2. Results (A - beta coefficients, B - p-values) of SSF run by individual at the 1 day scale, testing if selection takes a linear shape in the growing season.**

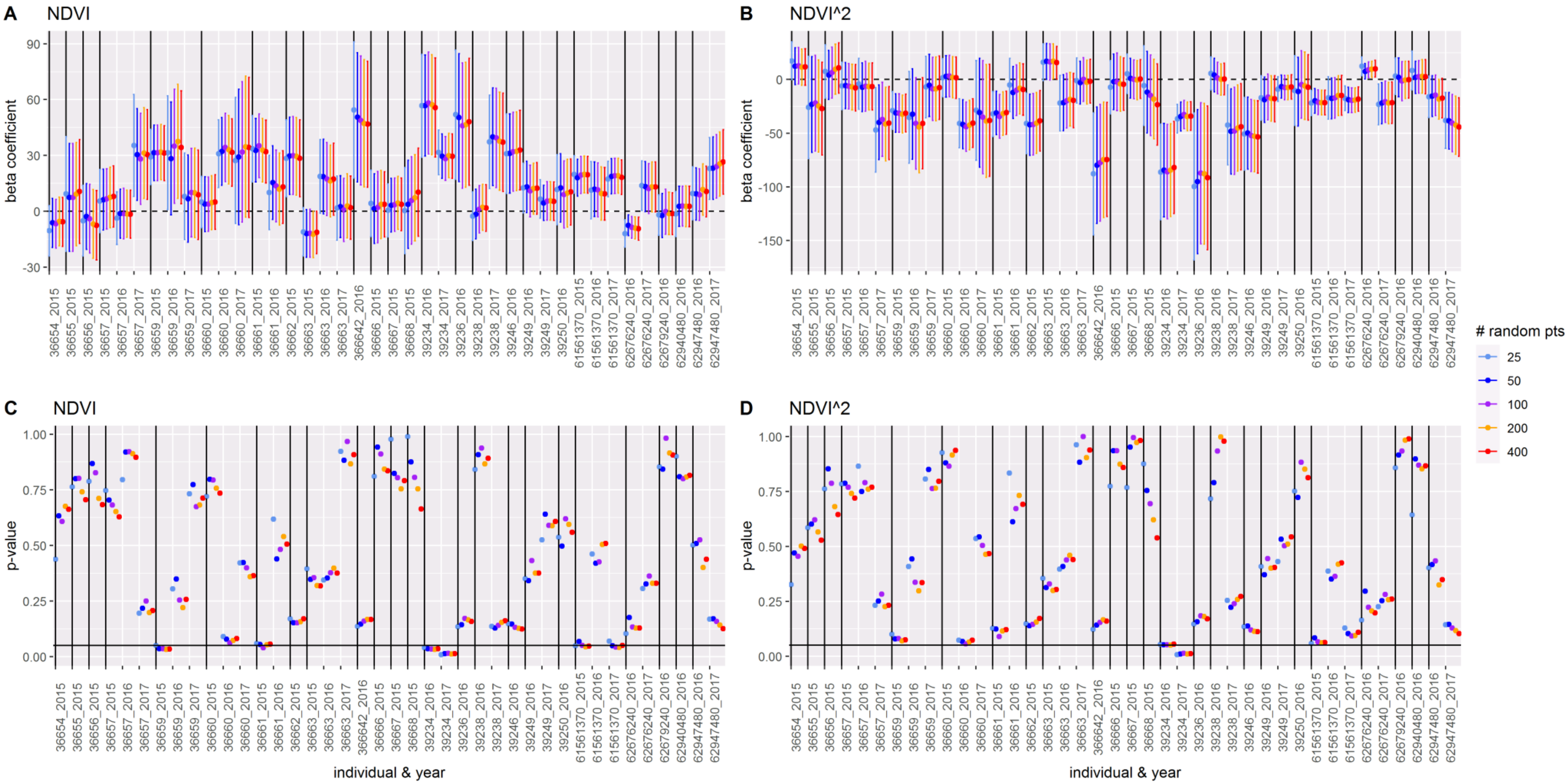


**Figure 3. Results (A,B - beta coefficients, C,D - p-values) of SSF run by individual/year at the 1 day scale, testing if selection takes a quadratic shape in the growing season.**

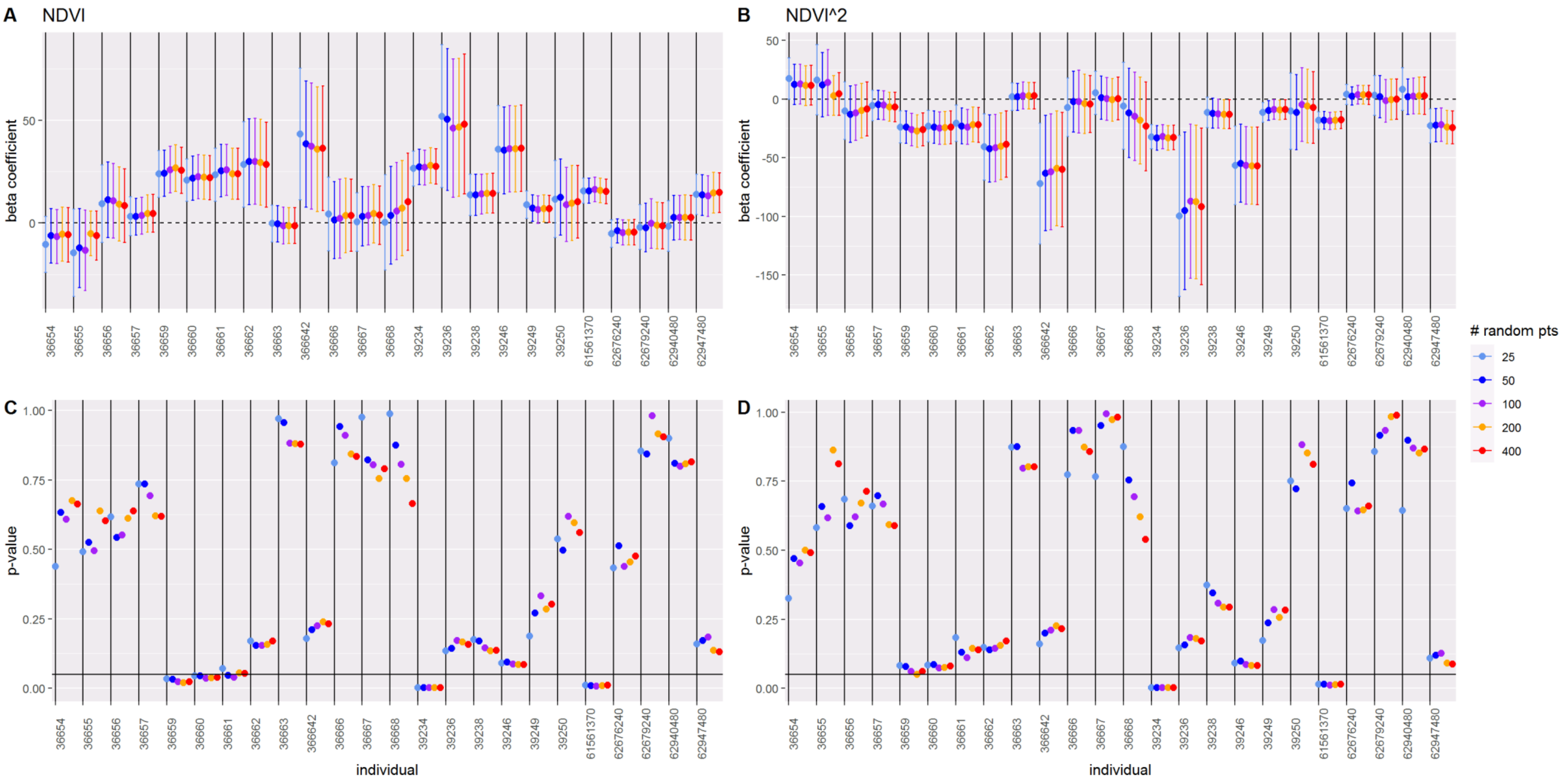


**Figure 4. Results (A,B - beta coefficients, C,D - p-values) of SSF run by individual at the 1 day scale, testing if selection takes a quadratic shape in the growing season.**

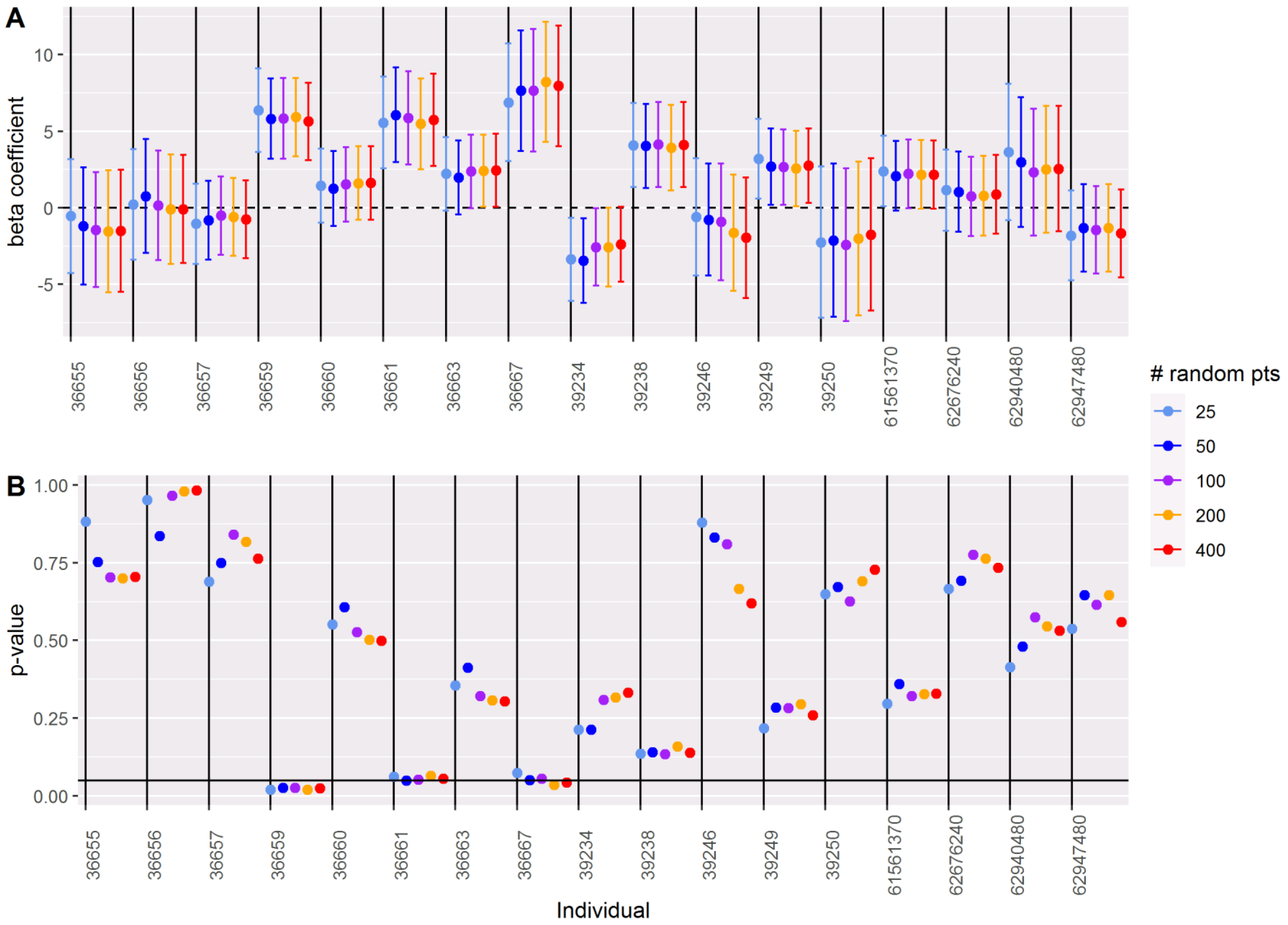


**Figure 5. Results (A - beta coefficients, B - p-values) of SSF run by individual at the 5 day scale, testing if selection takes a linear shape in the growing season.**

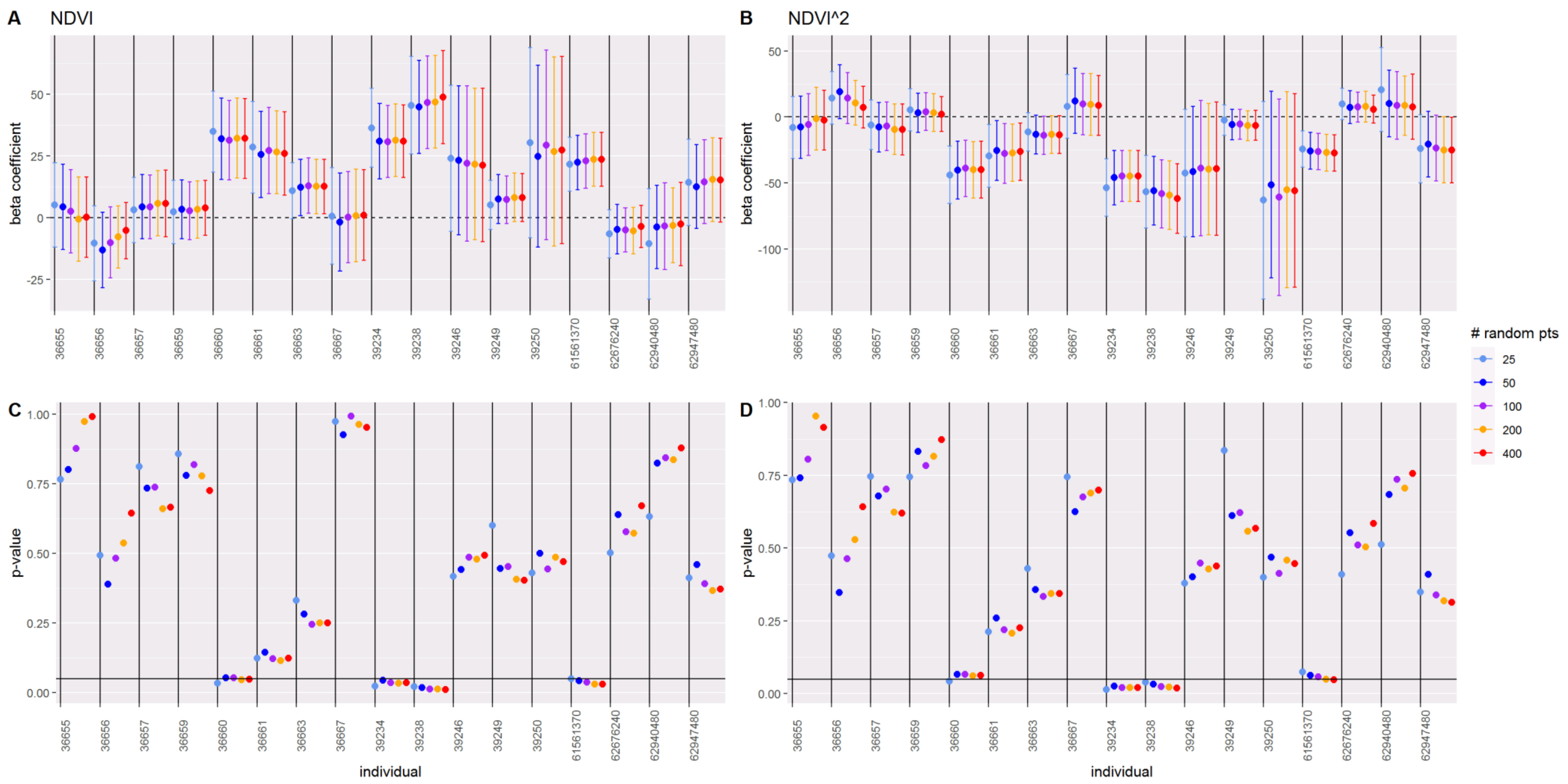


**Figure 6. Results (A,B - beta coefficients, C,D - p-values) of SSF run by individual at the 5 day scale, testing if selection takes a quadratic shape in the growing season.**

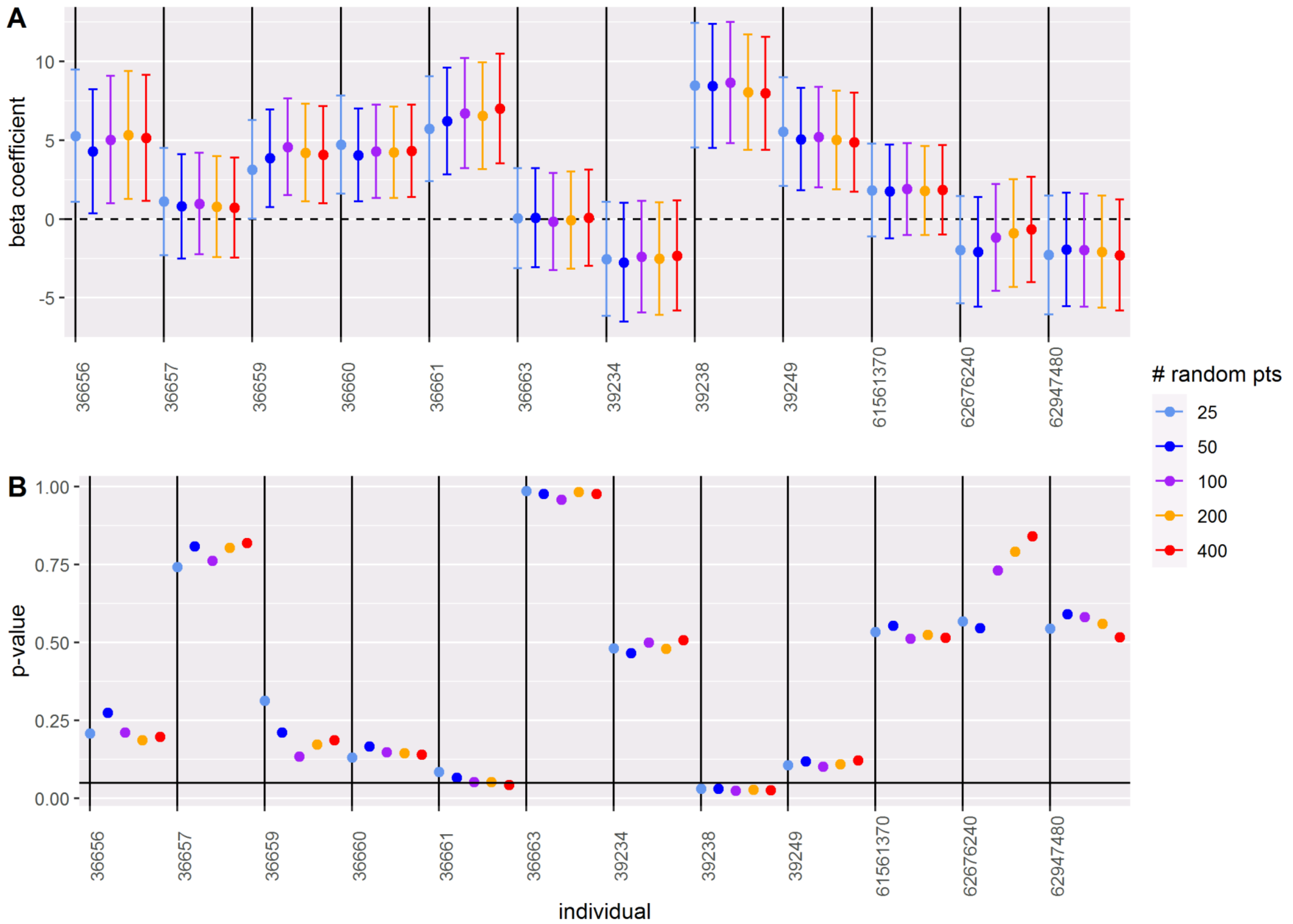


**Figure 7. Results (A - beta coefficients, B - p-values) of SSF run by individual at the 10 day scale, testing if selection takes a linear shape in the growing season.**

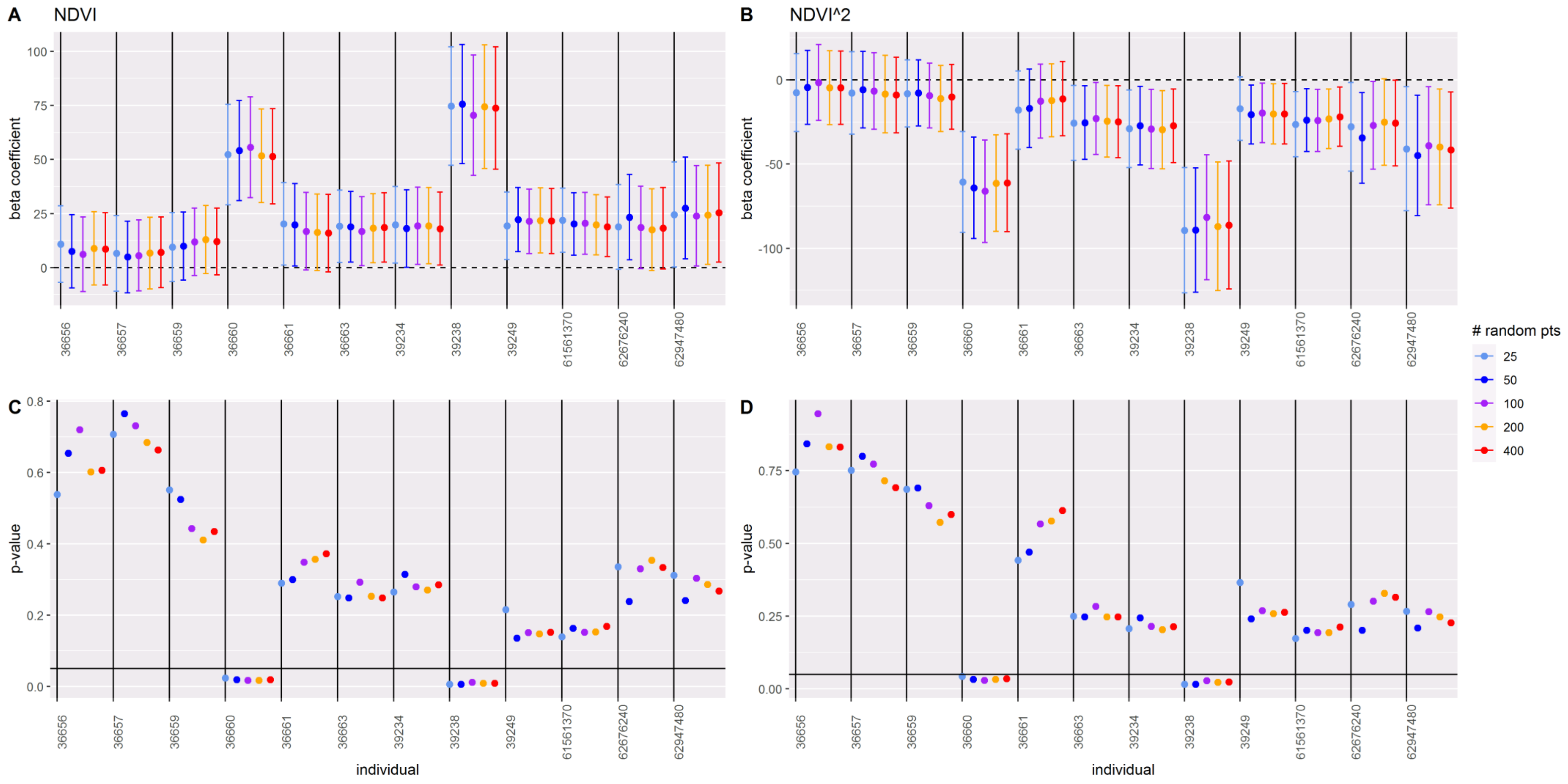


**Figure 8. Results (A,B - beta coefficients, C,D - p-values) of SSF run by individual at the 10 day scale, testing if selection takes a quadratic shape in the growing season.**

# Winter Results

Linear model: presence as a function of fraction snow cover in a 2 x 2 km area.
Quadratic model: presence as a function of snow cover + snow cover$^2$

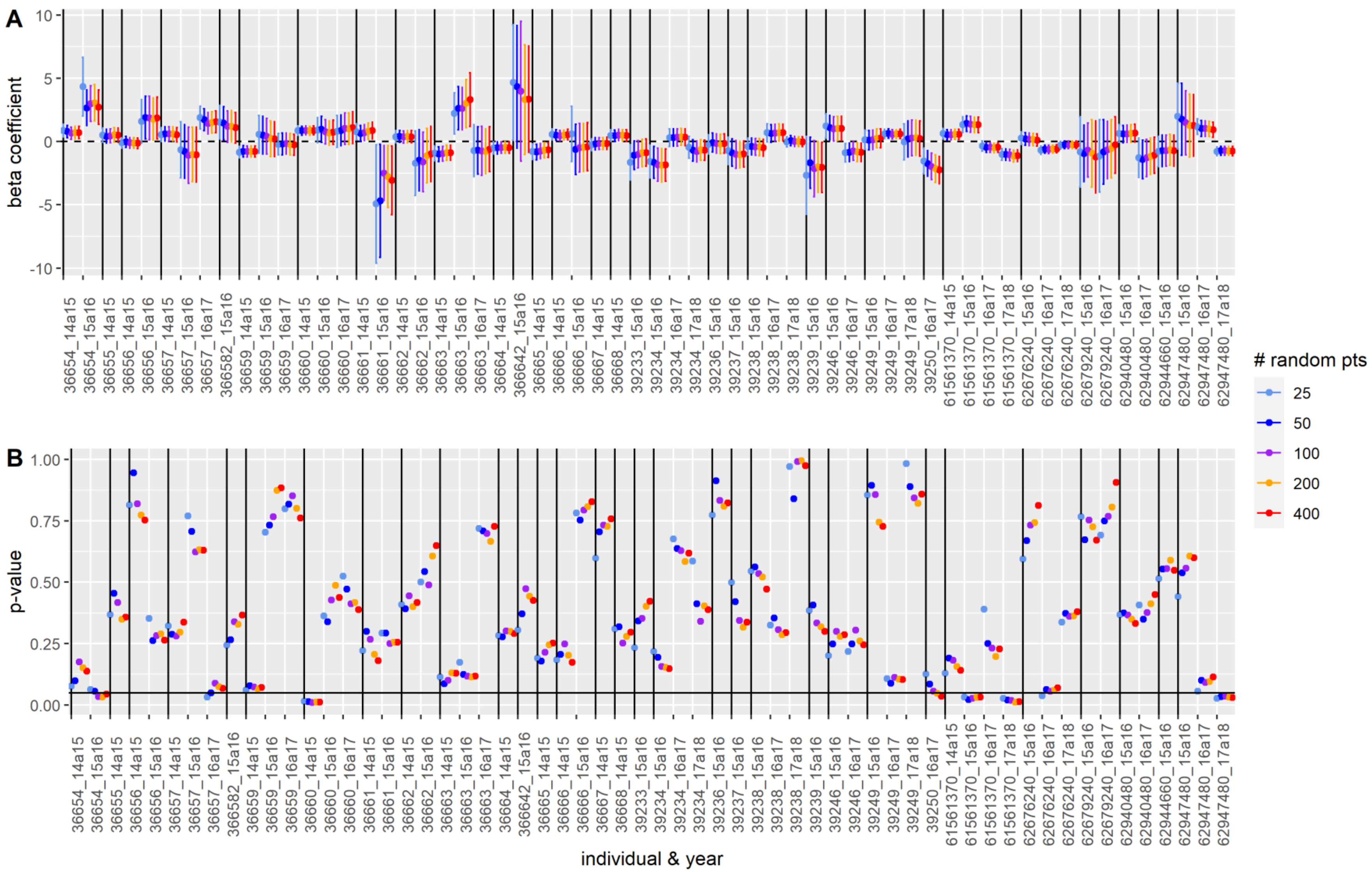


**Figure 9. Results (A - beta coefficients, B - p-values) of SSF run by individual/year at the 1 day scale, testing if selection takes a linear shape in winter.**

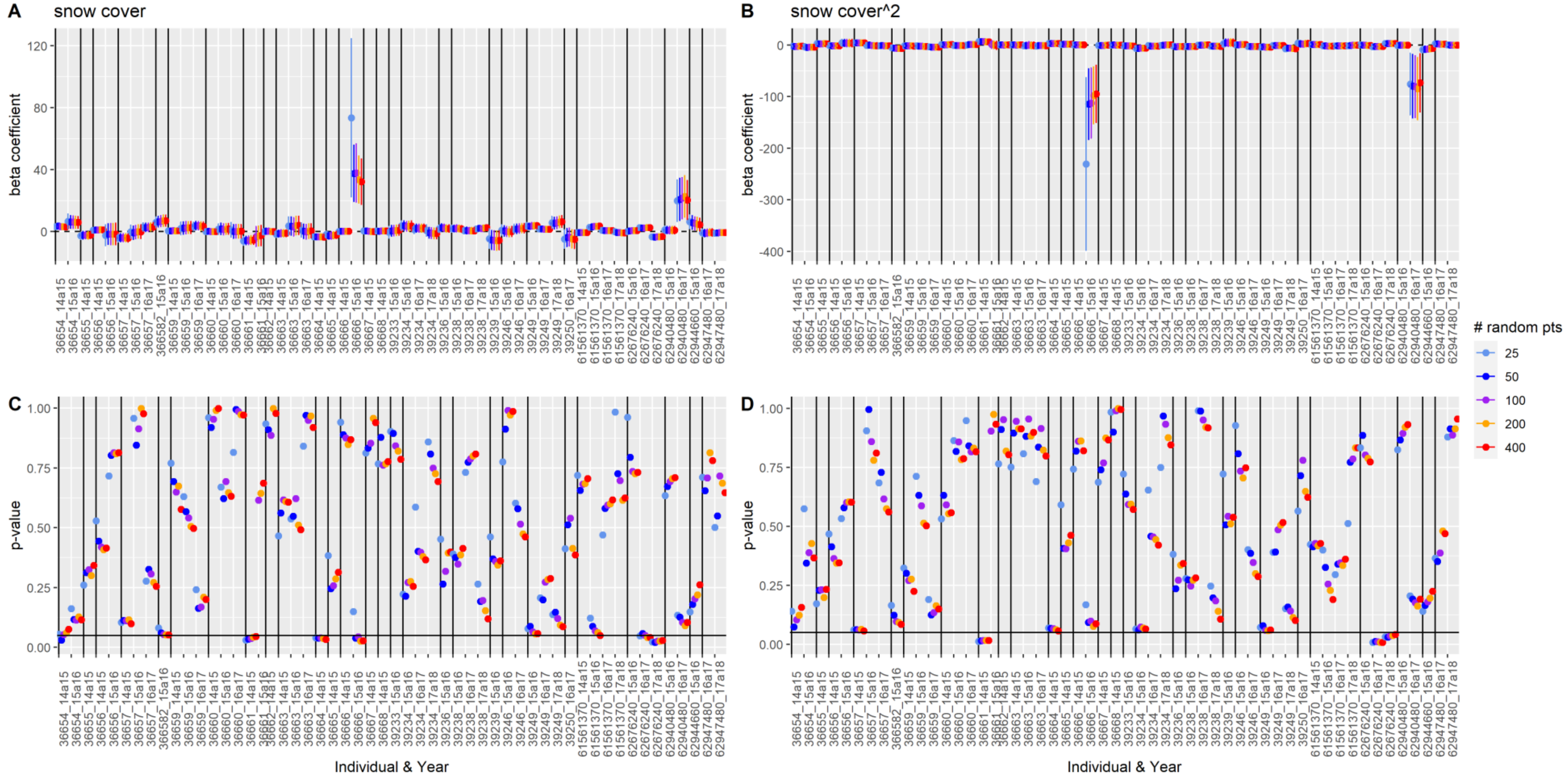


**Figure 10. Results (A,B - beta coefficients, C,D - p-values) of SSF run by individual/year at the 1 day scale, testing if selection takes a quadratic shape in winter.**

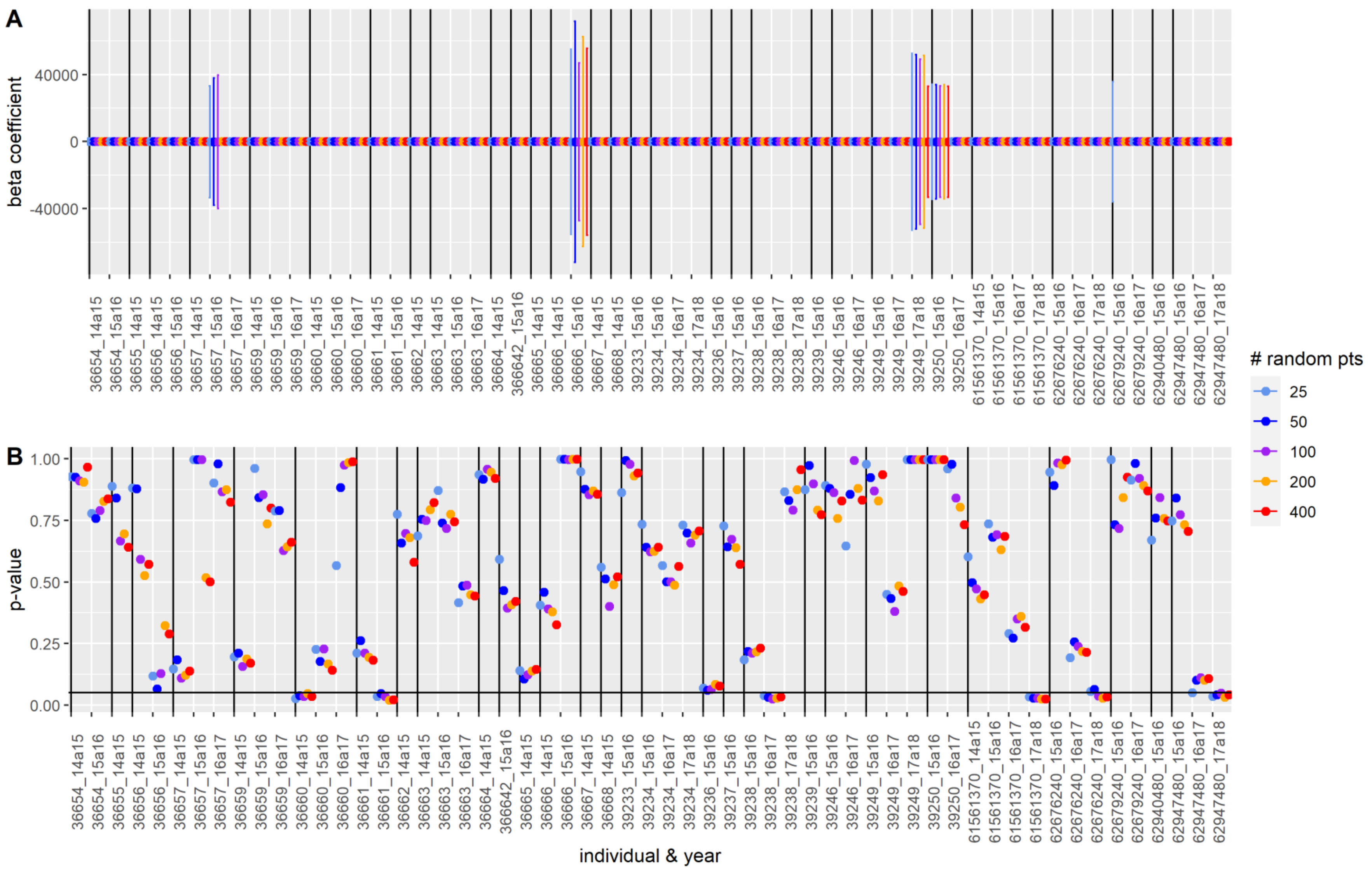


**Figure 11. Results (A - beta coefficients, B - p-values) of SSF run by individual/year at the 5 day scale, testing if selection takes a linear shape in winter.** Missing results for a random point value are due to high coefficient estimates that obscure the other results and were left out.

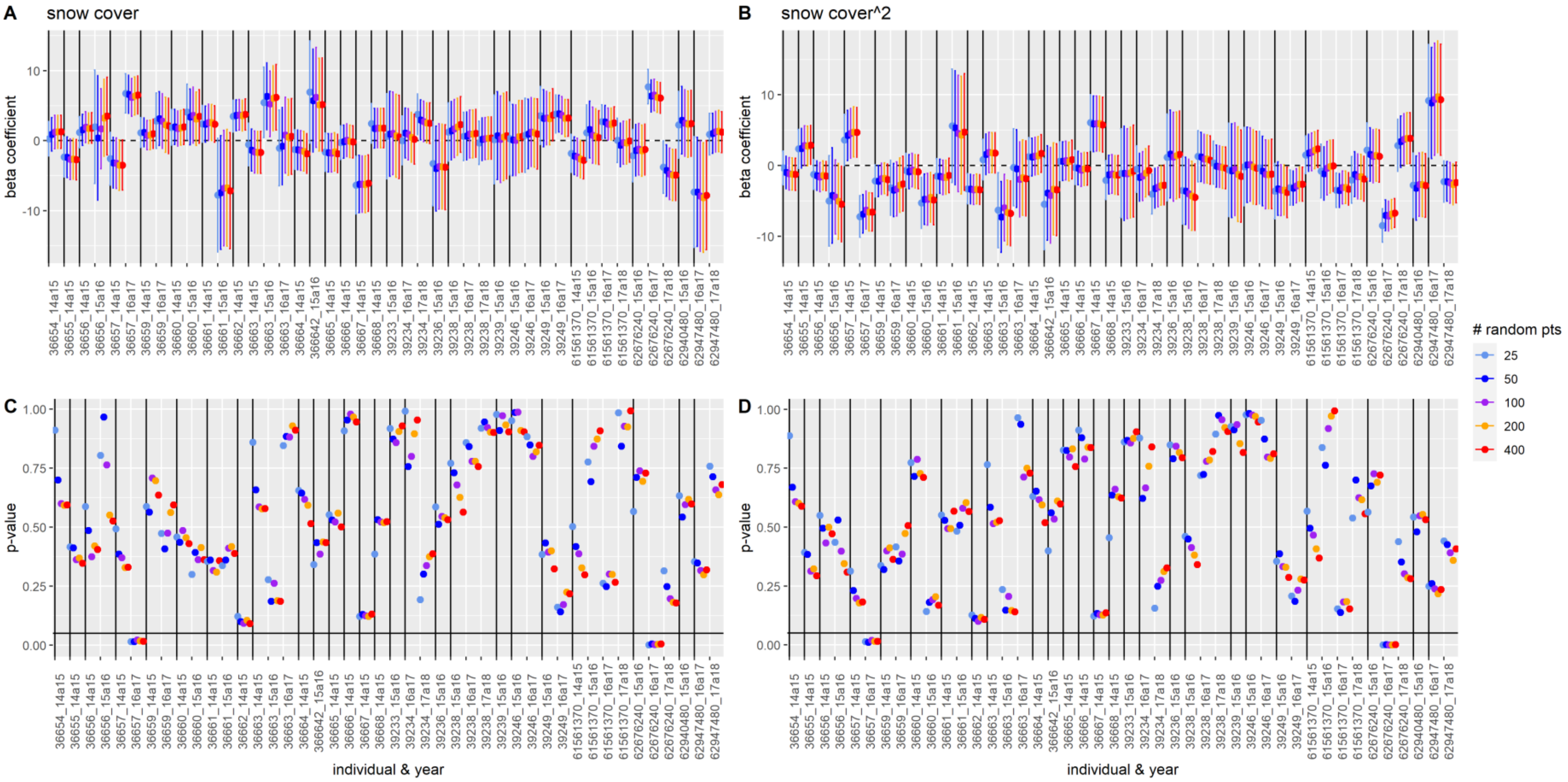


**Figure 12. Results (A,B - beta coefficients,  C,D - p-values) of SSF run by individual/year at the 5 day scale, testing if selection takes a quadratic shape in winter.** Missing results for a random point value are due to high coefficient estimates that obscure the other results and were left out.

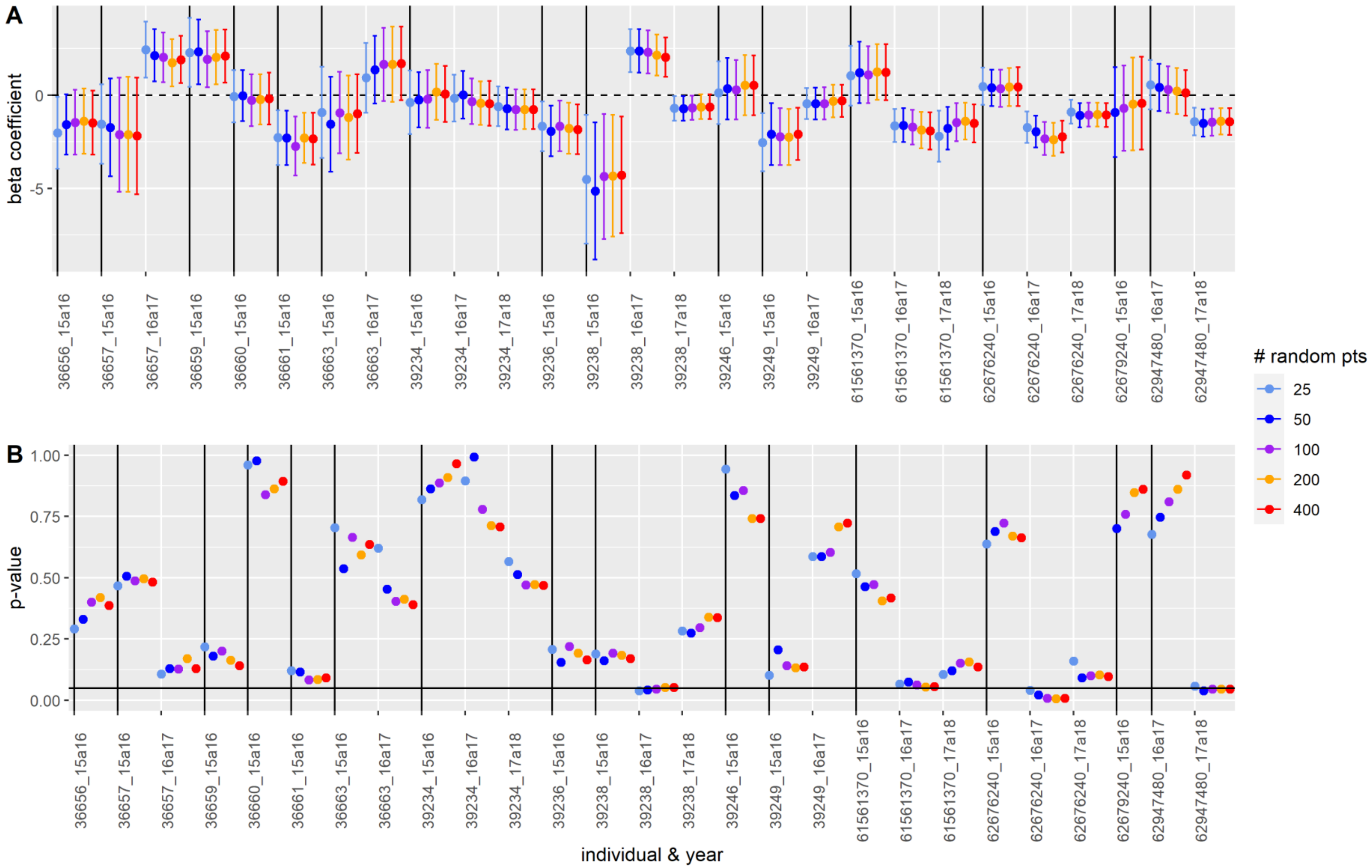


**Figure 13. Results (A - beta coefficients, B - p-values) of SSF run by individual/year at the 10 day scale, testing if selection takes a linear shape in winter.**

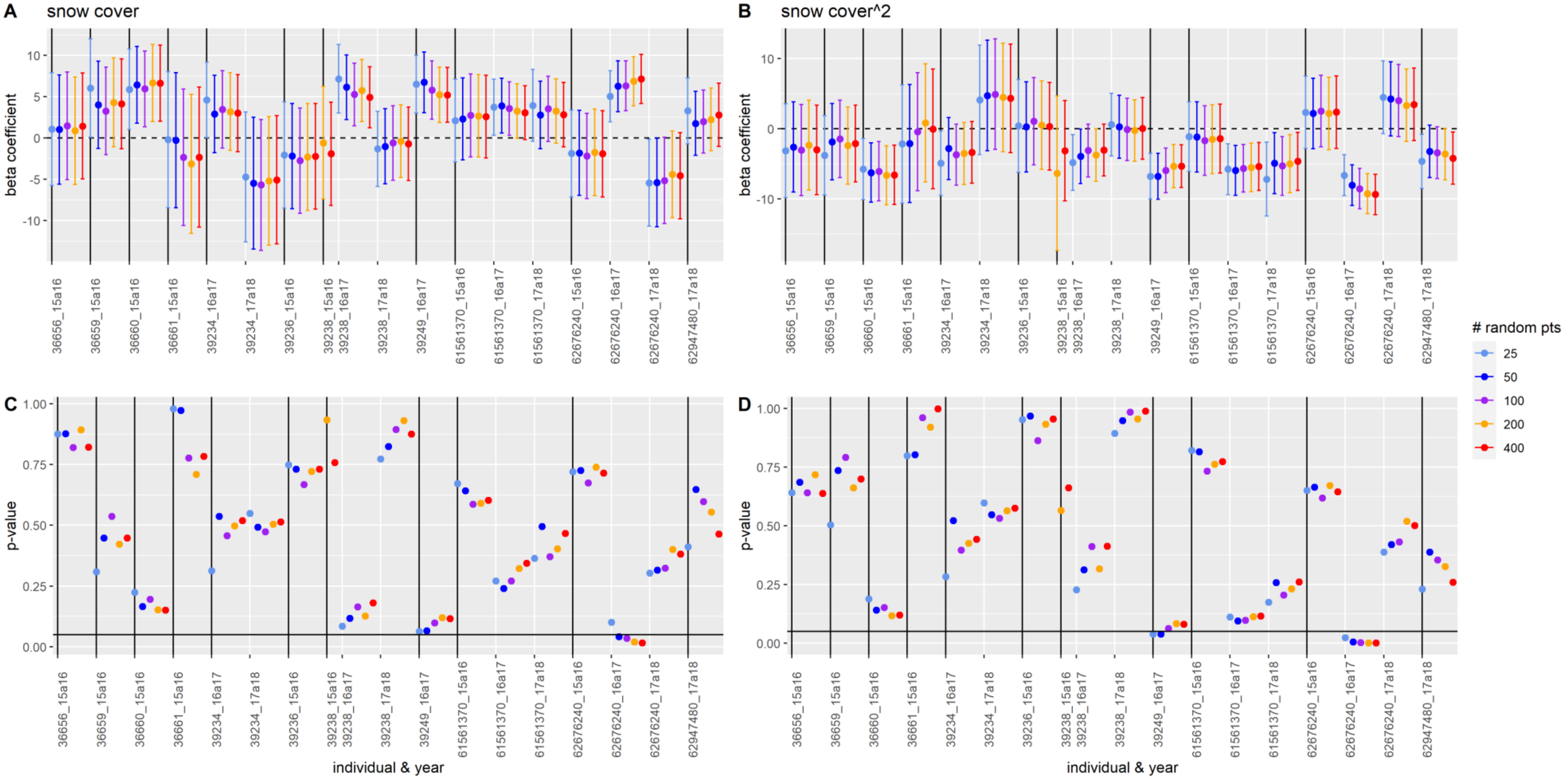


**Figure 14. Results (A,B - beta coefficients, C,D - p-values) of SSF run by individual/year at the 10 day scale, testing if selection takes a quadratic shape in winter.** Missing results for a random point value are due to high coefficient estimates that obscure the other results and were left out.
