# Supplementary material for: Resource selection of a nomadic ungulate in a dynamic landscape
Source: PLoS One. 2021 Feb 12;16(2):e0246809. doi: 10.1371/journal.pone.0246809 (PMC7880454; doi:10.1371/journal.pone.0246809)
Supplement: S2 Appendix — (PDF) [file pone.0246809.s019.pdf]

S2 Appendix: Additional analysis to look at differences in NDVI between areas selected by and available to Mongolian gazelles.

Landscape Characteristics

To examine how the landscape might influence our ability to detect selection at the finer scale of the SSFs we compared NDVI at gazelle locations to the surrounding landscape. First for each year from 2015-2017 we randomly drew 500 points used by gazelles during the growing season. Then we created buffers of increasing size around these points. We used the mean 1, 5, 10, and 15 day step-lengths of the gazelle population as the radii for the different buffers (1 day = 5km, 5 day = 17km, 10 day = 27 km, and 15 day = 35 km). For the 5, 10, and 15 day buffers we removed the area already covered by the other buffers, creating rings (1 day buffer = 0-5km from the used point, 5 day buffer = 5-17 km, 10 day buffer = 17-27km, and 15 day buffer = 27-35 km). Since the directional persistence decreases with increasing step size, the shape of these buffers reflects the fact that random points in the SSFs come from all directions around the presence point. We then drew 400 random points within these rings and extracted NDVI values to the random and presence points, using the NDVI scene corresponding to the time at which the gazelle was at that location. Then we compared the difference between the NDVI at the visited point and the mean NDVI of the surrounding area. We expected the differences to increase as the surrounding area moves further out.

Results

To understand why we did not detect selection in most individuals using the SSFs we compared NDVI at locations chosen by gazelles to their surrounding area. While we did observe a slight increase in the difference as the comparison area moved further out, in general the differences between used points and their surroundings were usually less than 0.05, even 15 days out (Fig 1). The mean difference at all scales was close to zero. Interestingly, box plots extended above and below zero, indicating that gazelles choose areas with lower or higher NDVI than the surrounding area about equally. Only in May do gazelles generally select for NDVI higher than the surrounding.

Discussion

A SSF compares the habitat at a presence point to points the gazelle could have reached but did not go to. Since gazelles show increasingly less directional persistence as step-length increases, these comparison points generally surround the presence point in all directions. If the mean habitat of these comparison points is very similar to the habitat at the presence point a SSF would interpret the data as the Mongolian gazelle not showing selection, even though it might be in high quality resources. In Figure 1 we see that a daily step (~ 5km), even a 15 day step (~35km) places the Mongolian gazelle within very similar habitat as seen by the SSF (Fig 1). This might explain why we found very few individuals showing selection.

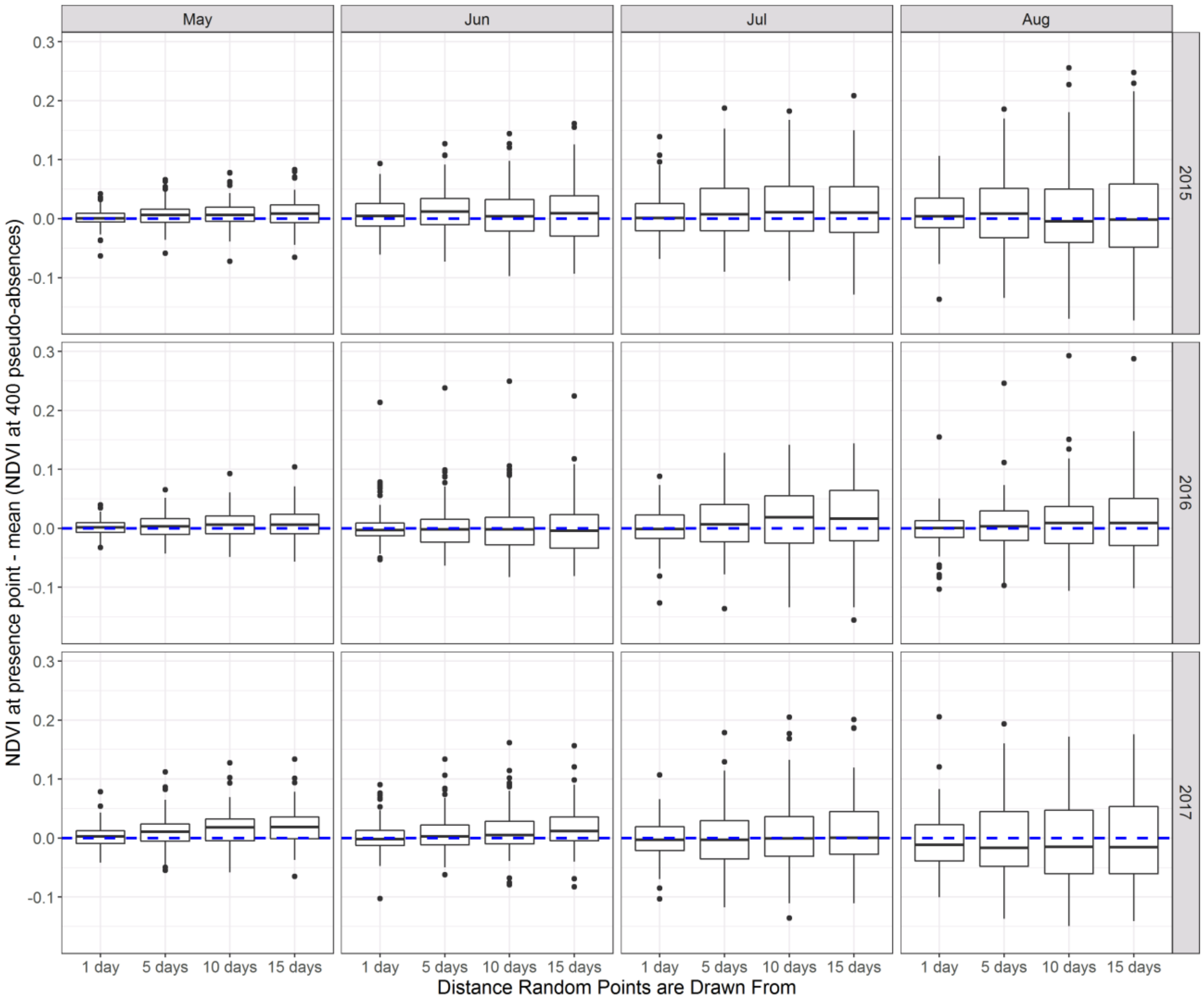

Figure 1. Areas used by Mongolian gazelles are very similar to their surroundings. Boxplots show the difference between NDVI at a point used by gazelles and mean NDVI of the surrounding area. Four different ring-shaped or circular buffers were created to define the surrounding area: a 1 day buffer 0-5km from the used location, a 5 day buffer 5-17km from the used location, a 10 day buffer 17-27km from the used location, and a 15 day buffer 27-35km from the used location. Differences between used areas and their surroundings remained small (<0.05), even when the surrounding area was 27-35km away.
