# Supplementary material for: Resource selection of a nomadic ungulate in a dynamic landscape
Source: PLoS One. 2021 Feb 12;16(2):e0246809. doi: 10.1371/journal.pone.0246809 (PMC7880454; doi:10.1371/journal.pone.0246809)
Supplement: Attachment — Submitted filename: PONE-D-20-30912_reviewer.pdf [file pone.0246809.s020.pdf]

PLOS ONE

Resource selection of a nomadic ungulate in a dynamic landscape

--Manuscript Draft--

**Manuscript Number:**	PONE-D-20-30912
**Article Type:**	Research Article
**Full Title:**	Resource selection of a nomadic ungulate in a dynamic landscape
**Short Title:**	Resource selection of a nomadic ungulate in a dynamic landscape
**Corresponding Author:**	Theresa Sonja Margareta Stratmann, MSc<br>Goethe University Frankfurt: Goethe-Universitat Frankfurt am Main<br>Frankfurt am Main, GERMANY
**Keywords:**	resource selection functions; step-selection functions; Procapra gutturosa; ungulate; nomadic; forage maturation hypothesis; Normalized Difference Vegetation Index; snow cover; Mongolia; rangeland
**Abstract:**	Nomadic movements are often a consequence of unpredictable resource dynamics. However, how nomadic ungulates select dynamic resources is still understudied. Here we examined resource selection of nomadic Mongolian gazelles (<i>Procapra gutturosa</i>) in the Eastern Steppe of Mongolia. We used daily GPS locations of 33 gazelles tracked up to 3.5 years. We examined selection for forage during the growing season using the Normalized Difference Vegetation Index (NDVI). In winter we examined selection for snow cover which mediates access to forage and drinking water. We studied selection on the population level using resource selection functions (RSFs) as well as on the individual level using step-selection functions (SSFs) at varying spatio-temporal scales from 1 to 10 days. Results from the population and the individual level analyses differed. At the population level we found selection for higher than average NDVI during the growing season. This may indicate selection for areas with more forage cover within the arid steppe landscape. In winter, gazelles selected for intermediate snow cover, which may indicate preference for areas which offer some snow for hydration but not so much as to hinder movement. At the individual level, in both seasons and across scales, we were not able to detect selection in the majority of individuals, but preferences were similar to those seen in the RSFs for those individuals showing selection. Difficulty in finding selection with SSFs may indicate that Mongolian gazelles are using a random search strategy to find forage in a landscape with large, homogeneous areas of vegetation. The combination of random searches and landscape characteristics could therefore obscure results at the fine scale of SSFs. The significant results on the broader scale used for the population level RSF highlight that, although individuals show uncoordinated movement trajectories, they ultimately select for similar vegetation and snow cover.
**Order of Authors:**	Theresa Sonja Margareta Stratmann, MSc
	Nandintsetseg Dejid
	Justin Calabrese
	William Fagan
	Christen Fleming
	Kirk Olson
	Thomas Mueller
**Additional Information:**	
**Question**	**Response**
**Financial Disclosure**<br><br>Enter a financial disclosure statement that describes the sources of funding for the work included in this submission. Review	TSMS was funded by the MainCampus Stipendium der Stiftung Polytechnischen Gesellschaft Frankfurt am Main.<br>TSMS, TM, and ND were funded by the Robert Bosch Foundation.<br>TSMS, TM, ND, and KAO were funded by the German Federal Ministry of Education and Research (BMBF, 01LC1710A and 01LC1820A).

Powered by Editorial Manager® and ProduXion Manager® from Aries Systems Corporation

the submission guidelines for detailed requirements. View published research articles from *PLOS ONE* for specific examples.<br><br>This statement is required for submission and **will appear in the published article** if the submission is accepted. Please make sure it is accurate.<br><br>**Unfunded studies**<br>Enter: *The author(s) received no specific funding for this work.*<br><br>**Funded studies**<br>Enter a statement with the following details:<br>• Initials of the authors who received each award<br>• Grant numbers awarded to each author<br>• The full name of each funder<br>• URL of each funder website<br>• Did the sponsors or funders play any role in the study design, data collection and analysis, decision to publish, or preparation of the manuscript?<br>• **NO** - Include this sentence at the end of your statement: *The funders had no role in study design, data collection and analysis, decision to publish, or preparation of the manuscript.*<br>• **YES** - Specify the role(s) played.<br><br>* typeset	JMC was partially funded by the Center of Advanced Systems Understanding (CASUS) which is financed by Germany's Federal Ministry of Education and Research (BMBF) and by the Saxon Ministry for Science, Culture and Tourism (SMWK) with tax funds on the basis of the budget approved by the Saxon State Parliament.<br>JMC, CHF, and WFF were supported by NSF Grant IIBR 1915347.<br>The funders had no role in study design, data collection and analysis, decision to publish, or preparation of the manuscript.
**Competing Interests**<br><br>Use the instructions below to enter a competing interest statement for this submission. On behalf of all authors, disclose any competing interests that could be perceived to bias this work—acknowledging all financial support and any other relevant financial or non-financial competing interests.<br><br>This statement **will appear in the published article** if the submission is accepted. Please make sure it is accurate. View published research articles from *PLOS ONE* for specific examples.	The authors have declared that no competing interests exist.

Powered by Editorial Manager® and ProduXion Manager® from Aries Systems Corporation

**NO authors have competing interests**<br><br>Enter: *The authors have declared that no competing interests exist.*<br><br>**Authors with competing interests**<br><br>Enter competing interest details beginning with this statement:<br><br>*I have read the journal's policy and the authors of this manuscript have the following competing interests: [insert competing interests here]*<br><br>* typeset	
**Ethics Statement**<br><br>Enter an ethics statement for this submission. This statement is required if the study involved:<br><br>• Human participants<br>• Human specimens or tissue<br>• Vertebrate animals or cephalopods<br>• Vertebrate embryos or tissues<br>• Field research<br><br>Write "N/A" if the submission does not require an ethics statement.<br><br>General guidance is provided below. Consult the submission guidelines for detailed instructions. **Make sure that all information entered here is included in the Methods section of the manuscript.**	Mongolian gazelles were captured and outfitted with GPS collars based on a standard protocol that was approved by the Ministry of Environment and Green Development in Mongolia (licenses: 6/5621, 5/4275).

**Format for specific study types**<br><br>**Human Subject Research (involving human participants and/or tissue)**<br>• Give the name of the institutional review board or ethics committee that approved the study<br>• Include the approval number and/or a statement indicating approval of this research<br>• Indicate the form of consent obtained (written/oral) or the reason that consent was not obtained (e.g. the data were analyzed anonymously)<br><br>**Animal Research (involving vertebrate animals, embryos or tissues)**<br>• Provide the name of the Institutional Animal Care and Use Committee (IACUC) or other relevant ethics board that reviewed the study protocol, and indicate whether they approved this research or granted a formal waiver of ethical approval<br>• Include an approval number if one was obtained<br>• If the study involved *non-human primates*, add *additional details* about animal welfare and steps taken to ameliorate suffering<br>• If anesthesia, euthanasia, or any kind of animal sacrifice is part of the study, include briefly which substances and/or methods were applied<br><br>**Field Research**<br><br>Include the following details if this study involves the collection of plant, animal, or other materials from a natural setting:<br>• Field permit number<br>• Name of the institution or relevant body that granted permission	
**Data Availability**<br><br>Authors are required to make all data underlying the findings described fully available, without restriction, and from the time of publication. PLOS allows rare exceptions to address legal and ethical concerns. See the PLOS Data Policy and FAQ for detailed information.	Yes - all data are fully available without restriction

Powered by Editorial Manager® and ProduXion Manager® from Aries Systems Corporation

A Data Availability Statement describing where the data can be found is required at submission. Your answers to this question constitute the Data Availability Statement and **will be published in the article**, if accepted.<br><br>**Important:** Stating 'data available on request from the author' is not sufficient. If your data are only available upon request, select 'No' for the first question and explain your exceptional situation in the text box.<br><br>Do the authors confirm that all data underlying the findings described in their manuscript are fully available without restriction?	
**Describe where the data may be found in full sentences. If you are copying our sample text, replace any instances of XXX with the appropriate details.**<br><br>• If the data are **held or will be held in a public repository**, include URLs, accession numbers or DOIs. If this information will only be available after acceptance, indicate this by ticking the box below. For example: *All XXX files are available from the XXX database (accession number(s) XXX, XXX.).*<br>• If the data are all contained **within the manuscript and/or Supporting Information files**, enter the following: *All relevant data are within the manuscript and its Supporting Information files.*<br>• If neither of these applies but you are able to provide **details of access elsewhere**, with or without limitations, please do so. For example:<br><br>*Data cannot be shared publicly because of [XXX]. Data are available from the XXX Institutional Data Access / Ethics Committee (contact via XXX) for researchers who meet the criteria for access to confidential data.*<br><br>*The data underlying the results presented in the study are available from (include the name of the third party*	For conservation concerns it is not standard to publish the raw GPS data on animal locations, but all the data used to run the statistical analyses and re-produce the figures will be made available on Dryad when the manuscript has been accepted.

and contact information or URL).<br>• This text is appropriate if the data are owned by a third party and authors do not have permission to share the data.<br><br>* typeset	
Additional data availability information:	Tick here if the URLs/accession numbers/DOIs will be available only after acceptance of the manuscript for publication so that we can ensure their inclusion before publication.

Powered by Editorial Manager® and ProduXion Manager® from Aries Systems Corporation

Manuscript Click here to access/download;Manuscript;stratmann_et_al.docx

Resource selection of a nomadic ungulate in a dynamic landscape

Theresa S. M. Stratmann[1,2*], Nandintsetseg Dejid[2], Justin M. Calabrese[3], William F. Fagan[4], Christen H. Fleming[4,5], Kirk A. Olson[6], Thomas Mueller[1,2]

[1] Department of Biological Sciences, Goethe University, Frankfurt am Main, Germany

[2] Senckenberg Biodiversity and Climate Research Centre, Senckenberg Gesellschaft für Naturforschung, Frankfurt am Main, Germany

[3] Center for Advanced Systems Understanding, Görlitz, Germany

[4] Department of Biology, University of Maryland, College Park, Maryland, U.S.A.

[5] Smithsonian Conservation Biology Institute, National Zoological Park, Front Royal, Virginia, U.S.A.

[6] Mongolia Program, Wildlife Conservation Society, Ulaanbaatar, Mongolia

* Corresponding author

E-mail: theresastrat@gmail.com (TSMS)

Abstract

Nomadic movements are often a consequence of unpredictable resource dynamics. However, how nomadic ungulates select dynamic resources is still understudied. Here we examined resource selection of nomadic Mongolian gazelles (*Procapra gutturosa*) in the Eastern Steppe of Mongolia. We used daily GPS locations of 33 gazelles tracked up to 3.5 years. We examined selection for forage during the growing season using the Normalized Difference Vegetation Index (NDVI). In winter we examined selection for snow cover which mediates access to forage and drinking water. We studied selection on the population level using resource selection functions (RSFs) as well as on the individual level using step-selection functions (SSFs) at varying spatio-temporal scales from 1 to 10 days. Results from the population and the individual level analyses differed. At the population level we found selection for higher than average NDVI during the growing season. This may indicate selection for areas with more forage cover within the arid steppe landscape. In winter, gazelles selected for intermediate snow cover, which may indicate preference for areas which offer some snow for hydration but not so much as to hinder movement. At the individual level, in both seasons and across scales, we were not able to detect selection in the majority of individuals, but preferences were similar to those seen in the RSFs for those individuals showing selection. Difficulty in finding selection with SSFs may indicate that Mongolian gazelles are using a random search strategy to find forage in a landscape with large, homogeneous areas of vegetation. The combination of random searches and landscape characteristics could therefore obscure results at the fine scale of SSFs. The significant results on the broader scale used for the population level RSF highlight that, although individuals show uncoordinated movement trajectories, they ultimately select for similar vegetation and snow cover.

Introduction

In landscapes where resource availability is dynamic, animals often migrate [1]. When resources are both dynamic and unpredictable, start and end points for a migration no longer exist, and animals must respond more flexibly, often through nomadic movements [2-4]. While much is known about resource selection of migratory animals - those animals which move between fixed locations at predictable times - much less is known about resource selection of nomadic animals. That which is known is often confined to one or two season within a year. Yet drivers of movement may differ between seasons, especially in temperate zones when snow can alter resource selection [5-7]. To complicate matters, nomadic animals show great individual variability in movement trajectories within the same landscape, a pattern that contrasts with the behavior of many migratory animals that show movement patterns which are more consistent across individuals [4]. Whether this individual variation is a result of among individual differences in resource selection, or simply due to random searches for food, remains unknown. To examine drivers of resource selection across the year within nomadic animals we used Mongolian gazelles (*Procapra gutturosa*) as a case study.

Resource selection during the vegetation growing season

For ungulates the forage maturation hypothesis (FMH) describes how plant characteristics might interact to influence herbivores' selection for vegetation. The FMH states that herbivores should select for vegetation of intermediate biomass [8-13]. New vegetation is highly nutritious but low in quantity and thus time consuming to find and eat. Older vegetation is high in quantity, requiring little time to find and eat, but low in nutritional value. This is due to plants building up their cell walls with lignin and ramping up chemical defenses as they grow, making them more difficult for herbivores to digest. Selecting for intermediate biomass therefore

optimizes energy intake. Hindgut fermenters can better meet their energy requirements on lower quality vegetation than ruminants. Ruminants require time for their efficient digestion, whereas hindguts can compensate for low quality vegetation by eating large quantities that are quickly digested [14]. We would therefore expect the FMH to be especially relevant for a ruminant like a Mongolian gazelle [14]. To work for migrating animals the FMH was further developed into the Green Wave Hypothesis (GWH) [15-19] which hypothesizes that animals migrate over elevational or latitudinal gradients to take advantage of multiple peaks in forage quality. However, in landscapes such as range and drylands, in which nomadic ungulates search for unpredictable forage, the GWH may not apply, because green wave tracking works best when the gradient of vegetation quality lasts over a long time and moves in a predictable order rapidly across the landscape [20-21]. This is why in this manuscript we have focused on the FMH. Elk (*Cervus elaphus*) [13, 22], wildebeest (*Connochaetes taurinus*) [23], and Thomson's gazelles (*Gazella thomsoni thomsoni*) [24] have all been found to select intermediate vegetation biomass according to the FMH. Two studies have even found support for nomadic ungulates following the FMH, i.e., nomadic animals seem to be able to find locations with high quality forage while searching an unpredictably changing landscape [24-25]. For example, [24] found that nomadic Thompson's gazelles positioned themselves in areas of above-average energy gain during the wet season. In addition, a previous study on Mongolian gazelle [25] found evidence for the FMH based on presence-absence data from transect surveys.

Resource selection in winter

While the FMH and GWH describe how ungulates are expected to select for forage during the growing season, selection strategies must shift during winter. For ungulates in temperate climates cold temperatures and snow become key factors shaping resource selection.

Snow can increase the cost of movement, restrict access to forage, or even prevent movement altogether [6, 26]. On the other hand, snow can serve as a means to hydrate [27-28]. Cold temperatures and icy winds can also introduce trade-offs between habitat types that offer shelter from thermoregulatory stress and deep snow (e.g., dense forest) and those that provide good forage (e.g., open meadows) [7, 29-31]. While selection for habitat types differs by species, most studies on winter habitat selection of ungulates find avoidance of deep snow, especially in small ungulates [5, 7, 26]. For ungulates living along elevation or latitudinal gradients this means moving to the valleys or lower latitudes [5, 32]. For ungulates in open steppes snow fall is less predictable, so the ability to move to escape snow storms is likely important, especially since there are no forests to seek cover in [33]. Because forage quality is generally low in winter, resource selection should be shaped by the need to maintain access to forage and the need to keep energetic costs low. While many have studied resource selection of ungulates in winter, few studies have examined resource selection by nomadic ungulates in winter. Two previous studies have examined population level resource selection of Mongolian gazelles in winter and both found selection for high forage biomass and areas with a short snow cover duration or little snow cover [28,34].

Importance of examining individual selection

Past studies of resource selection have often focused on selection at the population level, that is, on the average individual. These studies pooled data or results across individuals to arrive at an overall conclusion about what resources a population selects for. Yet recently studies have found individual differences in habitat selection which is forcing researchers to consider the implications of differences on management practices [35-36]. Considering individual selection and examining differences among individuals is especially interesting when studying nomadic

ungulates because individuals often show uncoordinated movements with greatly varying trajectories [4, 37]. The cause of these uncoordinated movements is poorly understood and difficult to study. Potential explanations are that individuals use a random search strategy to cope with unpredictable resource dynamics or that individuals show varying selection strategies due to different physiological states. Yet to-date no one has looked at individual variation in resource selection for Mongolian gazelles.

Life history of Mongolian gazelles

Mongolian gazelle are small, approximately 30kg ungulates native to the steppes of Russia, Mongolia, and China. Most Mongolian gazelles occur in the Eastern Steppe of Mongolia, the area east of the Ulaanbaatar - Beijing railroad, north of the Gobi Desert, and south of the Siberian forests. It is estimated that there are around 800,000 gazelles [38] making them a species of least concern [39]. Mongolian gazelles are intermediate feeders, exploiting high quality grasses and forbs during the growing season, and surviving off of dead vegetation in the winter by increasing the surface area of the rumen [40-41]. This change in physiology is key to surviving in this highly seasonal environment where green vegetation is mainly present from June to September and the winter is long and harsh with temperatures dropping below -40°C. Unpredictability in vegetation dynamics and snowfall are thought to be key drivers of this species' nomadic movements.

For Mongolian gazelles nomadic movements can be described by several characteristics. Individual Mongolian gazelles do not return to specific locations, not even during calving time or winter [37]. Individuals will move with conspecifics, but they merge with and split from groups in a fission-fusion manner. Thus individuals which are found in the same herd on one date can have completely different movement paths in the following days to years [3, 37]. Average annual

range size for Mongolian gazelles is ~ 19,346 km^2 but individual annual range sizes differ by an order of magnitude (range 6,431 km^2 – 53,422 km^2) [37]. This individuality in movement motivated us to examine not just population, but also individual responses to vegetation and snow cover.

Research approach and hypotheses

To study resource selection of Mongolian gazelles we used an extensive data set of daily GPS locations of 33 individuals. During the growing season we associated this data with the Normalized Difference Vegetation Index (NDVI), a satellite based measure of vegetation greenness that has been linked to vegetation quality and has been used to quantify resource availability [42-45]. Usually studies use field based measures (e.g., biomass, nitrogen content) to test the FMH. However, given the size of the Eastern Steppe and the temporal duration of our study, such field measurements would not have been feasible. Few other studies have used NDVI to test the FMH [25,46]. However, since high NDVI values correspond to higher vegetation biomass [47-50] we believe it is justified to test the FMH using NDVI. According to the FMH herbivores should avoid high biomass vegetation, which corresponds to high NDVI values. They should also avoid low biomass vegetation which corresponds to low NDVI values. Therefore, the FMH would predict selection for intermediate NDVI [25, 51]. Since Mongolian gazelles are ruminants, we hypothesized that they should select for intermediate NDVI during the growing season. In winter, foraging is mediated by snow depth, which can prevent movement and access to forage for small ungulates. On the other hand, snow is required as a source of drinking water. Since data on snow depth is not available for Mongolia, we used MODIS snow cover to examine resource selection in winter. We therefore hypothesized that gazelles should select for areas with intermediate snow cover during the winter, providing access to both water and forage. Finally, we

were interested in comparing population and individual level selection and hypothesized that selection would be similar at the population and at the individual-level. Despite the unique paths individual Mongolian gazelles take, we expected that these different paths would nonetheless lead Mongolian gazelles to areas with similar characteristics because there is a unifying physiological need for high quality vegetation and areas with low snow cover. To test these hypotheses, we (i) used resource selection functions (RSFs) [52] to examine population-level selection at the home-range scale for NDVI during the growing season and snow cover in winter and (ii) step-selection functions (SSFs) [53] to examine individual selection for NDVI and snow cover at 1, 5, and 10 day step-lengths.

Methods

Study area

The Eastern Steppe of Mongolia is one of the largest intact temperate grasslands left in the world [41]. It is an area with no trees, open plains, rolling hills, and few standing or flowing sources of water. There are only a few human settlements and few paved roads in this area, although oil and mineral extraction is becoming more common. Fences are rare in this landscape, except 1) at the border to Russia and China and 2) on both sides of the railroad leading from Ulaanbaatar to Beijing, both of which are highly effective at stopping gazelle movement [37,54].

The location of good pastures for wild and domestic herbivores is unpredictable, changing based on rain, snow, and fire events [55-57], eliciting nomadic movements in both humans and Mongolian gazelles. Mean annual rainfall is around 200 mm [57] and temperatures range from over 30°C in the summer to -40°C in the winter [27]. It is thought that this harsh climate controls both wild and domestic herbivore populations more than forage limitation does [59]. Mongolian gazelles must therefore adapt to a wide range of conditions across the year, and accordingly we

split our analysis into two timeframes. Timeframes were defined based on the presence of snow cover because it alters the interpretation of NDVI. For the first timeframe, which we call the growing season, we chose only NDVI scenes during which no snow cover was recorded during the years 2014 to 2018. This timeframe is from May 9 to August 29 and was chosen to match the temporal resolution of the MODIS data sets. The remainder of the year, from August 30 to May 8, we call winter since it is the period when snow can fall. While we could have examined more than two timeframes by taking the gazelle's life history into account (e.g., calving season during the growing season & rutting season during winter) these timeframes would have been too small to have sufficient data.

Mongolian gazelle tracking data

Mongolian gazelles were captured in the Eastern Steppe of Mongolia using drive nets [60]. This method of capture follows a standard protocol that was approved by the Ministry of Environment and Green Development in Mongolia (licenses: 6/5621, 5/4275). In October of 2014 16 Mongolian gazelles were captured. Fifteen of these Mongolian gazelles were outfitted with Lotek GPS collars programmed to collect location data every 23 hours, and one Mongolian gazelle was outfitted with a solar-powered Sirtrack GPS collar programmed to collect location data every hour. Two Mongolian gazelles died within a month of capture and so their data were not used. In September of 2015, 19 Mongolian gazelles were captured. Fourteen Mongolian gazelle received the Lotek GPS collars collecting daily locations and 5 received the solar-powered Sirtrack GPS collars collecting hourly data. Seven of the 33 Mongolian gazelles were male and all individuals were adults. We analyzed data up to May 8, 2018, giving us over 3.5 years of data for some individuals. Data from hourly Mongolian gazelles were summarized as a daily average location, so all data analyses were based on daily location data.

Remote sensing data

We used NDVI to quantify resource availability during the vegetation growing season. We used the 250 meter, 16 day NDVI data (MOD13Q1) from Terra MODIS (version 6) and downloaded it from the LP DAAC (https://lpdaac.usgs.gov/) and LAADS (https://ladsweb.modaps.eosdis.nasa.gov/) databases using the MODIS package in R [61]. To quantify snow cover we used the 500 meter resolution data set on maximum snow cover extent during an 8 day period (MOD10A2) from Terra MODIS (version 6). We used the MODIS package to download the data from National Snow and Ice Data Center (https://nsidc.org/data/). Using ESRI ArcGIS 10.6 we reclassified the data set into three categories: snow, no snow, and no data. No data consisted of cells marked as: no decision, night, lake, ocean, cloud, lake ice, detector saturated, or fill. We then used the aggregate tool to create a data set of mean snow cover extent in a 5 x 5km area. Five kilometers was chosen because it is the average distance a gazelle walks in a day. We chose to create this coarser resolution data set because snow can affect mobility and we were interested in not just the snow cover at the recorded GPS point, but what the surrounding area was like. While snow depth would be the most informative measure to use, such a data set does not exist for the Eastern Steppe.

Resource selection functions

All data preparation and analyses for the RSFs and SSFs were conducted in R (R version 3.5.1 and 4.0.1, accessed 10 July 2018 and 22 June 2020) [62].

To examine resource selection at the population-level we ran generalized linear mixed-effects models (GLMMs) for each season, pooling the daily GPS points from all Mongolian gazelles. This analysis therefore reflects what the average Mongolian gazelle selects for. To conduct this analysis we had to generate a set of 'pseudo-absence' points to use as a comparison

for the visited points. To confine our pseudo-absence points spatially we created a 100% minimum convex polygon (MCP) around all daily points for each individual- season- year combination using the package 'adehabitatHR' [63]. We then cropped the MCPs to be within Mongolia. While the 100% MCPs include some areas the Mongolian gazelles did not visit, their high mobility would have nonetheless made these areas available to them. The 100% MCPs also allowed us to examine how Mongolian gazelles placed themselves within the larger landscape as a contrast to the SSFs which looked at a much more local scale. Our models were run on a seasonal basis and each season included data from several MODIS NDVI (n = 7 in the growing season; n = 16 in winter) or snow cover scenes (n = 14 in the growing season; n = 32 in winter), which summarize NDVI over a 16 day period and snow cover over an 8 day period. The coarser temporal resolution compensates for days on which data quality is poor due to cloud cover. We wanted to make sure the environmental data for our pseudo-absence points were also split evenly among the MODIS scenes. Therefore for each MODIS scene/individual gazelle combination we: 1) selected the locations of an individual gazelle that fell within the time period of that scene, 2) used the rgeos [64] and dismo [65] packages to buffer those points by 5km, which is the average distance a Mongolian gazelle moves in a day, and removed this area from the individual-season-year MCP, 3) used the spsample function from the R package sp [66-67] to generate a stratified random sample of pseudo-absence points for that scene within the remaining MCP area, 4) extracted the NDVI or snow cover values to the random points, and 5) repeated for each scene-individual combination. RSFs require a large number of random points [68] so we created data sets with 1, 2, 5, 10, and 20 times more pseudo-absence points than presence points. This allowed us to determine when model beta coefficients became stable. We then used the glmer function in the lme4 package [69] to run the GLMMs. To avoid biased estimates of the beta coefficients we gave presence points a weight of 1 and pseudo-absence points a weight of 1000 [70]. For each

season, we ran two models. The first model had a quadratic term for the fixed effect of either NDVI or snow cover (e.g., NDVI + $NDVI^2$) with random slopes and intercepts for each individual Mongolian gazelle and random intercepts for each year. A quadratic model can test whether individuals are selecting for intermediate resource values (downward parabola) or both the minimum and maximum available resource values (upward parabola). The second model had a linear term for the fixed effect, NDVI or snow cover, with random slopes and intercepts for each individual Mongolian gazelle and random intercepts for each year. This linear model tests whether Mongolian gazelles selected for the maximum resource value (positive slope) or the minimum resource value (negative slope). For each season we then used AIC to compare support for these four different hypotheses regarding the shape of selection (i.e., intermediate, extremes, maximum, or minimum). In addition to testing two models, we ran all models with an increasing number of pseudo-absence points. Coefficients did not change much after using 20 times the number of pseudo-absence points as presence points so we present results from these models (S1 Fig). In the growing season, the models were singular because year did not explain any variance in the data. We therefore re-ran the models without year as a random intercept. Models were still singular, because individual also explained little variance, and so we also ran the models without random effects using the glm function in the stats package [62]. In winter, year was also singular in the linear models, so we re-ran the models without a random intercept for year.

Step selection functions

To examine selection of individual Mongolian gazelles for NDVI we used step-selection functions (SSFs). SSFs use conditional logistic regression (clogit) to compare areas where Mongolian gazelles were found to areas where the animals could have gone, based on their step-lengths and relative turn angles [53]. SSFs group comparisons of a presence point and its

corresponding pseudo-absence points into one step. The pseudo-absence points represent locations that the Mongolian gazelle could have also reached in a given time step but did not go to. For each step the environment at the presence location is compared with the environment at the pseudo-absence locations. Doing this for many steps allowed us to determine if there was a significant difference in the environment between where a Mongolian gazelle was found and where it could have been. SSFs allow us to examine selection at finer scales. Since resource selection is scale dependent [71] the biological interpretation of SSFs depends on the interval between locations (the step-length) [72].

It is unknown at what scale Mongolian gazelles select for vegetation and snow cover. We therefore tested multiple scales. Usually, the vegetation (or snow cover) a day's walk away (~ 5 km displacement on average, based on our data) is very similar. We therefore also tested step lengths of 5 days (~17 km displacement on average) and10 days (~ 27 km displacement). This lets us sample a variety of spatial scales, but because sample size diminishes with each increase in step-length we were not able to test larger steps. To generate the data sets for the different scales we went through all the daily locations identifying sets of consecutive GPS locations with a minimum of three points per set. Three points is the minimum required to calculate a relative turn angle. We did the same for points 5 and 10 days apart. From these data we could then calculate 1, 5, and 10 day displacements (e.g., step-lengths) and relative turn angles. Data from all individuals were pooled to lessen the influence of unusual displacement lengths or low sample sizes for individuals with less data. Using these pooled data, we created 25, 50, 100, 200, and 400 pseudo-absence points for each point visited by the animal. NDVI or snow cover values were then extracted to the presence and pseudo-absence points. The appropriate MODIS scene to use was chosen by matching the date of the presence point to the NDVI scene it falls within.

SSFs were run on an individual-season-year basis (e.g., Mongolian gazelle 1 in the spring of 2015) for all three spatial scales (1, 5, and 10day step-lengths) for the winter models because mixed-effects models such as those proposed by [73] usually did not converge for our data set. Yet for the growing season models the 5 and 10 day step scales had too little data to split it by year, we therefor ran these models by individual and ran the 1 day models by both individual-year and individual as comparison. We used the 'clogit' function in the survival package [74-75] to run our models. We only kept individual-season-year or individual-season combinations that had at least 20 steps. For each individual-season-year dataset we fit a linear model (just NDVI or just snow cover) and a quadratic model (NDVI + NDVI^2 or snow cover + snow cover^2) to test the same hypotheses we tested in the RSFs. Within each season-year, we determined how many individuals showed significant selection ($\alpha \leq 0.05$) in either the linear or quadratic models. We used AIC to determine which hypothesis had more support, but we do not report on top models that were not significant as our hypotheses likely did not capture the underlying process well in these cases. Based on the top models we categorized selection into selection for the maximum (linear model with a positive slope), selection for the minimum (linear model with a negative slope), selection for intermediate values (quadratic model, downward parabola), or selection for extreme values (quadratic model, upward parabola). Since there is no good rule of thumb for the number of required pseudo-absences, models were run with 25, 50, 100, 200, and 400 pseudo-absence points to examine when beta coefficients became stable. With 400 random points beta coefficients no longer changed much (S1Appendix). While p-values still changed, this was only for the non-significant models. Significant models generally showed stable p-values across the range of pseudo-absence points (S1 Appendix). Results are therefore presented for model runs with 400 pseudo-absence points.

To visualize our data we plotted relative selection strength against available NDVI or snow cover [76]. Relative selection strength acknowledges that selection is always dependent on a choice between two or more options. Because we were interested in the FMH we chose mean NDVI for a given year as our comparison point for the growing season models by individual-year. For the models by individual, we chose mean NDVI during the 2015-2017 growing seasons. For the winter models we used mean snow cover for a given year because we also hypothesized that gazelles would select for intermediate snow cover. The mean value was calculated by pooling both the presence and pseudo-absence data within a season from all individuals, but dividing it by year for the yearly means. Because these values show some extremes due to a few points falling in very rare habitats, we restricted the data by removing the upper and lower 5% of NDVI (snow cover) values. The relative selection strength curves show selection strength for NDVI (snow cover) values available to an individual in a given season-year if that individual must choose between those values or the mean NDVI (snow cover) value. Available NDVI (snow cover) is the range of NDVI (snow cover) covered by the points the individual Mongolian gazelle visited and by the associated pseudo-absence points. Again, we restricted the available values by removing the upper and lower 5% of NDVI (snow cover) values. A characteristic of the plotting method we used is that different reference points result in different selection strengths, but the NDVI (snow cover) value at which selection peaks stays the same. For comparisons between individuals, across seasons and years we therefore focused on the NDVI (or snow cover) value at peak selection and not selection strength.

Results

On the population level, in both seasons, quadratic models offered a better fit than linear models (Table 1). During the growing season gazelles selected for intermediate NDVI (β_{NDVI}=

6.97, $p < 0.001$; $\beta_{NDVI}{}^2$= -7.97, $p < 0.001$; S1 Table). The preferred NDVI value (0.44, the NDVI value at which log(RSS) peaks) was above the average NDVI value within the population range over the three growing seasons (0.30) (Fig 1). In fact, the preferred value was at the upper end of the NDVI available to gazelles (0.17 – 0.49). We defined available NDVI to exclude NDVI values in the upper and lower 5% of the data. Compared to the maximum and minimum NDVI observed (-0.1925 and 0.8116 respectively) the preferred NDVI value would be more intermediate, although still above the mean which remains 0.30 for the entire data set. During winter we found significant selection for intermediate snow cover values at the population level (Fig 1; β_{snow}= 1.59, $p < 0.001$; $\beta_{snow}{}^2$= -1.80, $p < 0.001$; S2 Table). While our focus was on the population level result (i.e. the average individual), the random effects explained considerable variation, mostly through the random slopes for individual (S3 Table; marginal $R^2 = 0.002$, conditional $R^2 = 0.62$). The preferred snow cover value was just below the mean snow cover within the population range over the four winters.

Table 1. AIC values for the different growing season (May 9 – Aug. 29) and winter (Aug. 30 – May 8) RSF models examining how Mongolian gazelles select for NDVI and snow cover.

Season	Hypothesis	df	AIC	Weight	ΔAIC
Growing Season	Quadratic	3	73554	0.66	0.00
	Linear	2	73587	<0.001	33.1
Winter	Quadratic	7	212706	1	0
	Linear	4	212997	<0.001	291

Models represent different hypotheses on the shape of selection (linear or quadratic) for NDVI during the growing season and for snow cover during winter. During the growing season, the models have no random slope for individual or random intercepts for individual and year because even simplified random effects resulted in singular fits. For winter, the quadratic model has a random slope for individual and random intercepts for individual and year. The linear model has no random intercept for year because this produced a singular fit.

Fig 1. Selection by Mongolian gazelles for A) snow cover during winter and B) NDVI during the growing season. RSFs show Mongolian gazelles select for intermediate snow cover in winter (Aug. 30 – May 8). During the growing season (May 8 – Aug. 29), gazelles select for above average NDVI**.** Selection is quantified as relative selection strength (RSS). Curves were plotted based on the best fit RSF for the population. The vertical line indicates the mean NDVI (fraction snow cover), which was used as the reference point to create the curves. Dots indicate the most preferred NDVI (fraction snow cover) value compared to the mean NDVI (fraction snow cover). The curve is only plotted for the range of NDVI (fraction snow cover) observed at the used and available points over the study period (Oct. 2014- May 2018), where the upper and lower 5% of values have been removed. Means are also based on this subset.

In contrast to the population level analyses, the individual SSFs were not informative. Few individuals showed significant selection at any scale (Fig 2). If sample size were a problem, we would expect to find many more individuals selecting at the 1 day scale then at the 5 and 10 day scale, but this was not the case (see S4-S6 Tables for sample sizes). For the growing season, we did not have sufficient data to run individual-year models for the 5 and 10 day scales, so we report on results from models run by individual at all scales (but see S2 and S3 Figs and S7 Table for the 1 day SSFs run by individual-year). For those individuals who did show selection during the growing season the results were similar to the RSFs, with individuals selecting for above average NDVI, often at the upper end of available NDVI, at all scales (Fig 3; S4 and S5 Figs). In the SSFs, though, this was the result of selection taking a quadratic or linear shape (Fig 3; S7 Table). In winter, we had enough data to look at selection by individuals within a year. Gazelles selected for the maximum, minimum, and intermediate amount of snow cover available to them within a year (Fig 4; S6-S8 Figs and S5 Table). This meant that individuals selected snow cover both above and below the mean snow cover during a given year. This mix of selection types and

a lack of consistency were seen at all scales (S7 and S8 Figs). We also examined if gazelles that had more than one year of data showed the same selection across years. While most gazelles showed consistent selection, up to a quarter did switch selection strategies (1 day scale = 6 individuals out of 19 with more than one year of data, 5 day scale = 5 out of 17, 10 day scale = 4 out of 7, Fig 5). Switching selection could also include switching between selecting and not selecting.

Fig 2. Differences among Mongolian gazelles in selection for NDVI during the growing season and for snow cover in winter at different scales (A – 1 day, B – 5 day, C – 10 day step scale) based on individual SSFs. Blue and yellow sections of the bar plots show the percent of individuals selecting for NDVI during the growing season or for snow cover during winter, grey shows the percent of individuals that did not show selection. For those individuals showing selection, dark blue indicates selection for the highest NDVI (or snow cover) available, light blue indicates selection for the lowest NDVI (or snow cover) available, and yellow selection for intermediate values. Above each bar are the number of individuals which had sufficient data (minimum of 20 steps) to run step-selection functions. The data show no evidence for selection for the majority of individuals and this trend holds across all scales tested.

Fig 3. Selection for NDVI at the 1 day step scale shown by individual Mongolian gazelles during the growing season (May 9 – Aug. 29). Selection is quantified as relative selection strength (RSS). Curves were plotted based on the results of the individual SSFs and show the best fit model for an individual. Only those individuals who showed significant selection at the 1 day step-scale are shown. The vertical line indicates the mean NDVI (as described in the methods), which was used as the reference point to create the curves. Dots indicate the most preferred NDVI values compared to the mean NDVI. The curve is only plotted for the range of NDVI

available to the individual gazelle as defined in the methods. Results for the 5 and 10 day scales can be found in the supplementary materials.

Fig 4. Selection for snow cover at the 1 day step scale shown by individual Mongolian gazelles in a given year during winter (Aug. 30 – May 8). One individual is not shown for clarity, but see S2 Fig. Selection was quantified as relative selection strength (RSS). Curves were plotted based on the results of the individual SSFs and show the best fit model for an individual. Only those individuals who showed significant selection at the 1 day step-scale are shown. The vertical line indicates mean snow cover (as described in the methods), which was used as the reference point to create the curves. Dots indicate the most preferred snow cover value compared to mean snow cover. The curve is only plotted for the range of snow cover available to the individual gazelle as defined in the methods. Results for the 5 and 10 day scales can be found in the supplementary materials.

Fig 5. Percent of Mongolian gazelles that showed different selection strategies for snow cover in different years according to SSFs. Not all Mongolian gazelles show consistent selection strategies for snow cover across years. For all individual gazelles for which we had more than one year of data during the winter season the percent that switched their selection strategy at least once was calculated. This could be a switch from one type of selection to another (e.g. high snow cover vs. intermediate snow cover) or from selecting to not showing selection. We did not have sufficient data to run individual-year models at all scales during the growing season.

Discussion

Selection at the population level

We explored resource selection of nomadic Mongolian gazelles across years and seasons at the population scale and at the individual, daily to weekly scale. During the growing season, at the population level, selection took a quadratic shape with the average gazelle selecting for above average NDVI. We therefore found only limited support for our hypothesis that gazelles select for intermediate NDVI according to the FMH. Here we emphasize that while the preferred value of NDVI is an intermediate value of NDVI, which ranges from -0.2 to 1, it is above the mean available NDVI and in the upper range of the available NDVI. NDVI describes vegetation greenness and the observed values in the steppe were generally less than 0.5. Lower NDVI values can reflect areas with more visible bare soil [43, 77-78]. Such areas partially occur naturally in the steppe, but are also due to drought or overgrazing by livestock [47, 79]. Therefore gazelles might be choosing areas with higher NDVI because these have denser vegetation cover and thus more forage. Therefore with NDVI we might not be able to detect for selection for forage quality according to the FMH, but the observed selection suggests that gazelles find areas with higher forage cover. Yet at some point preference for higher NDVI begins to decrease again, likely because these areas reflect tall, dense vegetation that is difficult to digest and serves as a hiding place for predators such as wolves.

In winter the population selected for intermediate snow cover. This supports our hypothesis that in winter gazelles avoid areas of high snowfall, but seek out areas that provide enough snow for hydration without restricting movement or access to forage.

Previous work on Mongolian gazelles found selection for intermediate NDVI at the population level in spring and fall [25]. In winter, two separate studies both found avoidance of areas with snow, especially those with a long snow-cover-duration [28, 34]. Discrepancies between these studies and ours could be methodological in origin. [25] and [34] used presence/absence data from line transect surveys to calculate RSFs, while [28] used GPS data

from 8 Mongolian gazelles to build a Maxent habitat suitability model. Despite their differences these studies and ours all indicate that nomadic animals can adjust to unpredictable vegetation during the growing season and find vegetation free from too much snow cover in winter.

Comparison of population and individual level results

Whereas previous studies have focused on selection for forage at the population level, we also examined selection at the individual level. The motivation for this individual level analysis was that Mongolian gazelles captured at the same place and time often exhibit distinct movement paths that take them to very different areas of the steppe. We were interested if individuals nonetheless end up in areas with similar vegetation or snow cover or if individuals show differences that might then be an explanation for these unique movement paths. In other studies that ran both RSFs and SSFs the focus was to examine changes in selection at different scales (e.g., selection of the home range vs. selection within the home range) [80-81]. Our hypothesis, though, was that selection for vegetation quality and snow cover should be similar at both scales (i.e., within the population seasonal home range and along the individual movement path) and among individuals despite their different movement paths. This is because unlike other landscapes that are composed of numerous land cover classes, our landscape simply comprises one habitat class: grassland. While a deer may choose forest for its home range and open meadows within the forest to forage in, such heterogeneity of habitat classes does not exist in the Mongolian steppe. What varies is the quality of the grasses and forbes due to growing state or plant composition and the depth of the snow. Currently there is no readily available remote sensing data to examine these aspects of the steppe. Therefore our interest in testing our hypothesis at the population and individual scale was to account for the individualistic movement paths which are more defined in the SSFs than the RSFs. While we found patterns in selection at

the population level and can describe the selection of an average gazelle, our results from the SSFs examining individual level selection were inconclusive. For most individuals in both seasons we could not detect selection. During the growing season, the few individuals for which we were able to detect selection showed a preference for NDVI above the mean across all scales, which is consistent with the results of the RSFs. The main difference is that selection took both a linear and a quadratic shape. Therefore some individuals showed selection according to the FMH, selecting NDVI in the intermediate range of what was available to them, although NDVI at peak selection was still above the mean of available NDVI. In winter, individuals selected for high, low, and intermediate snow cover and so they selected for snow cover both above and below the mean of available snow cover. This variability was seen at all scales. Individuals that selected for high snow cover might have gotten stuck in deep snow, or snow depth was too low to impact movement and forage. Our inability to detect selection in the majority of individuals was surprising. If sample size (see S6-S8 Table) were a problem we would have expected to see many more individuals showing selection at the 1 day scale than at the larger scales. While there was a decrease, it was minimal since so few individuals already showed selection at the 1 day scale. There are several possible explanations, both methodological and biological, which could explain these results. These explanations are discussed below and may shed light on aspects of SSFs that have received little attention yet and should be considered in future research.

The role of landscape configuration and random searches

One explanation for our inability to detect selection at the individual level using SSFs may be the homogeneity of the landscape across very broad scales. NDVI in the steppe changes only across broad scales which may lead to small differences of gazelle locations and their surroundings within a daily or weekly movement scale (S2 Appendix) [3]. SSFs reduce available

resources to those within the selected step scale, which users should carefully choose [72], but which often is determined by the frequency of the GPS fixes. While gazelle cover large distances at the 5 and 10 day scales (27-35km), NDVI variability at these distances may have still been too small to show any significant differences in NDVI between selected and random locations (S2 Appendix). If NDVI at selected and random points are similar, then SSFs would interpret this as no selection, even if the gazelle is in an area with high quality forage. This implies that while Mongolian gazelles may make decisions at sub-daily time scales on which plants to eat based on their digestibility, when it comes to finding the larger area within which they should forage they need to move far before they are in an area with different characteristics. This raises the question of what search strategies can be used when a landscape has large, homogeneous patches. If gazelles were to use perception, this would require perception of resources beyond their sight. The Eastern Steppe is a flat, treeless landscape. In similar landscapes in the Serengeti wildebeest (*Connochaetes taurinus*) were observed moving toward a thunderstorm they heard 24 kilometers away or toward rainstorms that darkened the sky as far as 80 kilometers away [82]. In addition, Mongolian gazelles which have found good resources could potentially call to other gazelles several kilometers away to share that information [83]. Therefore gazelles might be able to find good resource patches, even if they are far away.

Alternatively, given the large patch sizes of vegetation and their unpredictable dynamics using perception might not, or only partially, be feasible, and gazelles may in many cases use random search strategies to find new resources. This is because in unpredictable resource landscapes, memory and gradient following are not expected to be useful in finding resources. Instead, animals might employ area restricted searches [84-85] that may be less efficient than perception and which might often place Mongolian gazelles in unfavorable habitat and thus make it difficult to detect selection in general.

Whether gazelles use random searches or use perception to choose between large, homogeneous habitat patches – selection for foraging patches is likely to occur only rarely. If this is the case then SSFs might be too fine-scaled to pick up on these rare events. Step-lengths can only be increased to a certain point before sample size becomes limiting or the area examined becomes similar to an RSF approach. Using SSFs with nomadic animals may therefore require using integrated step-selection functions to associate changes in movement (e.g., speed, turn angle) with differences in resource quality [86]. Although, we would still face the challenge of finding a step-length that is appropriate for both the behavior of the animal and the composition of the landscape. For both SSFs and RSFs another useful approach would be to categorize movement trajectories into foraging and searching behavior which would allow for a more focused examination of selection during certain behavioral modes [80, 87-88]. This might also help us identify over what scales gazelles forage and search for forage. SSFs could then be used within foraging patches to look at vegetation preferences, but this would require sub-daily data and better data on vegetation quality. Yet these categorization methods have not been tested on nomadic animals. Finally, by simulating random searches of a theoretical animal with a known habitat preference, future work could examine what approach is best for studying resource selection in animals that use random search strategies.

Selection in dynamic landscapes

An additional factor that may complicate the SSF approach is the possibility that resource configuration in the Mongolian steppe may change over time. By using NDVI the spatial configuration of resources changes over space and time. How homogeneous or heterogeneous the landscape is will influence at what scale animals can select for vegetation and at what scale SSFs can identify selection. If the configuration of resources in space constantly changes, then SSFs,

which can only test one scale at a time, might have difficulty in detecting selection. Other studies across a number of taxa have used NDVI for SSFs (Canada lynx [*Lynx canadensis*] – [80]; pronghorn [*Antilocapra americana*] – [89]; Hartmann's mountain zebra [*Equus zebra hartmannae*] – [90]; African elephants [*Loxodonta africana*] – [91]), but no study indicated similar challenges with NDVI. All found selection for NDVI, although the time steps examined were much smaller than in our study (<= 4hrs). Smaller time steps result in more data that might have made it easier to detect selection, given the environment is sufficiently heterogeneous to distinguish selected from available points. For all studies NDVI was only one of many variables tested and we do not have enough information about the dynamics and configuration of the respective landscapes to draw comparisons to our study. Future work could use simulations of landscapes and animal movements to examine how landscape configuration influences SSFs' ability to detect selection.

Variable selection

For those individuals where SSFs detected significant selection, the direction and degree of selection for NDVI was variable and inconsistent. Across years individuals showed different selection patterns. Consistent differences in selection among individuals are not unusual and are often associated with animal personalities [35]. In such cases, it is important that individuals display the same selection patterns across many years. Because our results did not indicate consistency across years, they are difficult to interpret. Yet one explanation could be that differences in selection among individuals or differences within individuals across years might be due to an individual's physiological state. For example, reproductive state, sex, age, thermal stress, changing energy requirements, and health have all been shown to affect selection strategies of animals [30, 92-94]. Individual gazelles may therefore have different selection

strategies depending on their current state which could cause differences among individuals as well as differences within individuals across years. Thus, in some cases it may be beneficial to maximize energy intake by selecting for intermediate biomass vegetation. In other cases, selecting high biomass vegetation can allow an individual to meet its basic energy requirements quickly, leaving time for other activities such as reproduction [30, 95-96]. In addition, seasonal and patchy presence of biting insects, human activity, the need to protect calves, droughts, and heat waves could all alter selection strategies for longer time periods.

Unexamined drivers of selection

Another simple explanation for the inconclusive SSF results might be that other factors are driving selection at a local scale. Wolves, biting flies, human disturbance, and livestock grazing likely all alter how gazelles select for vegetation. Because the presence of all these factors changes in space and time they are difficult to measure and we were not able to account for them. Yet, [46] showed that although there are fewer Mongolian gazelles in areas of intermediate NDVI with more humans, selection for intermediate NDVI is still detectable. Thus, we would expect additional biotic and abiotic factors to weaken but not eliminate our ability to detect selection for vegetation or snow cover. It might have also been easier to detect selection if data on the digestibility or composition of the vegetation was available for the growing season and data on snow depth was available for winter. NDVI and snow cover are the best available metrics that are available across time and at broad scales, but are only rough proxies for habitat quality.

Conclusion

Our conclusions on how nomadic Mongolian gazelles track vegetation resources and snow cover are defined by the population-level RSF. Only at this scale were we able to detect

selection for NDVI and snow cover. Despite the unpredictable landscape and unique movement trajectories, the RSFs show Mongolian gazelles do select areas with similar vegetation during the growing season. Therefore there appears to be similarity in selection among individuals. While we would need data on the digestibility or species composition of the vegetation to understand why gazelles selected for higher than average NDVI values, it is striking that nomadic individuals tracking unpredictable vegetation nonetheless find similar areas of vegetation. Similarly, in winter, the population showed preference for intermediate snow cover. Interestingly, we were not able to detect selection for most individuals using SSFs. This may bring attention to aspects of SSFs that yet need to be studied and we hope our study inspires future research into the how landscape configuration and search strategy may influence the performance of SSFs which in turn would help to better understand resource selection of herbivores in grassland systems. This would be especially important for nomadic animals where SSFs could help us better understand the unique movement paths that individuals take and if they are related to fine-scale resource selection.

Acknowledgements

We would like to thank Jörg Albrecht and Johannes Signer for their statistical insight and assistance. It would not have been possible to collect the data on Mongolian gazelles without the help of numerous field assistants, the World Wide Fund for Nature (WWF Mongolia), which helped to obtain the animal capture permits, and the Ministry of Environment and Tourism of Mongolia.

References

1. Alerstam T, Hedenström A, Åkesson S. Long-distance migration: evolution and determinants. Oikos. 2003 Nov 1;103(2):247–60.
2. Mueller T, Fagan WF. Search and navigation in dynamic environments – from individual behaviors to population distributions. Oikos. 2008 May 1;117(5):654–64.
3. Mueller T, Olson KA, Dressler G, Leimgruber P, Fuller TK, Nicolson C, et al. How landscape dynamics link individual- to population-level movement patterns: a multispecies comparison of ungulate relocation data. Glob Ecol Biogeogr. 2011 Sep 1;20(5):683–94.
4. Teitelbaum CS, Mueller T. Beyond Migration: Causes and Consequences of Nomadic Animal Movements. Trends Ecol Evol. 2019 Jun 1;34(6):569–81.
5. Mysterud A, Bjørnsen BH, Østbye E. Effects of snow depth on food and habitat selection by roe deer Capreolus capreolus along an altitudinal gradient in south-central Norway. Wildl Biol. 1997 Mar;3(3/4):27–33.
6. Robinson BG, Merrill EH. The influence of snow on the functional response of grazing ungulates. Oikos. 2012;121(1):28–34.
7. Gilbert SL, Hundertmark KJ, Person DK, Lindberg MS, Boyce MS. Behavioral plasticity in a variable environment: snow depth and habitat interactions drive deer movement in winter. J Mammal. 2017 Feb 8;98(1):246–59.
8. van Soest PJ. Nutritional ecology of the ruminant. Corvallis: O and B Books; 1982.
9. McNaughton SJ. Grazing Lawns: Animals in Herds, Plant Form, and Coevolution. Am Nat. 1984;124(6):863–86.
10. McNaughton SJ. Grazing Lawns: On Domesticated and Wild Grazers. Am Nat. 1986;128(6):937–9.

11. Hobbs NT, Swift DM. Grazing in Herds: When are Nutritional Benefits Realized? Am Nat. 1988;131(5):760–4.

12. Fryxell JM. Forage Quality and Aggregation by Large Herbivores. Am Nat. 1991 Aug 1;138(2):478–98.

13. Hebblewhite M, Merrill E, McDermid G. A multi-scale test of the forage maturation hypothesis in a partially migratory ungulate population. Ecol Monogr. 2008 May 1;78(2):141–66.

14. Illius AW, Gordon IJ. Modelling the nutritional ecology of ungulate herbivores: evolution of body size and competitive interactions. Oecologia. 1992 Mar 1;89(3):428–34.

15. Drent R, Ebbinge B, Weijand B. Balancing the energy budgets of arctic-breeding geese through- out the annual cycle: a progress report. Verhandlungen Ornithol Ges Bayern. 1978;23:239–64.

16. Owen M. Wild geese of the world: their life history and ecology. London: B. T. Batsford Ltd.; 1980.

17. Bischof R, Loe LE, Meisingset EL, Zimmermann B, Moorter Bram Van, Mysterud A. A Migratory Northern Ungulate in the Pursuit of Spring: Jumping or Surfing the Green Wave? Am Nat. 2012 Oct 1;180(4):407–24.

18. Armstrong JB, Takimoto G, Schindler DE, Hayes MM, Kauffman MJ. Resource waves: phenological diversity enhances foraging opportunities for mobile consumers. Ecology. 2016 May 1;97(5):1099–112.

19. Merkle JA, Monteith KL, Aikens EO, Hayes MM, Hersey KR, Middleton AD, et al. Large herbivores surf waves of green-up during spring. Proc R Soc B. 2016 Jun 29;283(1833):20160456.

20. Aikens EO, Kauffman MJ, Merkle JA, Dwinnell SPH, Fralick GL, Monteith KL. The greenscape shapes surfing of resource waves in a large migratory herbivore. Ecol Lett. 2017;20(6):741–50.

21. Aikens EO, Mysterud A, Merkle JA, Cagnacci F, Rivrud IM, Hebblewhite M, et al. Wave-like Patterns of Plant Phenology Determine Ungulate Movement Tactics. Curr Biol. 2020 Jul;S0960982220308484.

22. Wilmshurst JF, Fryxell JM, Hudsonb RJ. Forage quality and patch choice by wapiti (Cervus elaphus). Behav Ecol. 1995 Dec 1;6(2):209–17.

23. Wilmshurst JF, Fryxell JM, Farm BP, Sinclair A, Henschel CP. Spatial distribution of Serengeti wildebeest in relation to resources. Can J Zool. 1999 Nov 1;77(8):1223–32.

24. Fryxell JM, Wilmshurst JF, Sinclair ARE. Predictive models of movement by serengeti grazers. Ecology. 2004 Sep 1;85(9):2429–35.

25. Mueller T, Olson KA, Fuller TK, Schaller GB, Murray MG, Leimgruber P. In search of forage: predicting dynamic habitats of Mongolian gazelles using satellite-based estimates of vegetation productivity. J Appl Ecol. 2008 Apr 1;45(2):649–58.

26. Parker KL, Robbins CT, Hanley TA. Energy Expenditures for Locomotion by Mule Deer and Elk. J Wildl Manag. 1984;48(2):474–88.

27. Jiang Z, Takatsuki S, Gao Z, Jin K. The present status, ecology and conservation of the Mongolian gazelle, Procapra gutturosa : a review. Mammal Study. 1998;23(1):63–78.

28. Ito TY, Sakamoto Y, Lhagvasuren B, Kinugasa T, Shinoda M. Winter habitat of Mongolian gazelles in areas of southern Mongolia under new railroad construction: An estimation of interannual changes in suitable habitats. Mamm Biol. 2018 Nov 1;93:13–20.

29. Mysterud A, Østbye E. Cover as a Habitat Element for Temperate Ungulates: Effects on Habitat Selection and Demography. Wildl Soc Bull 1973-2006. 1999;27(2):385–94.

30. Beest FM van, Milner JM. Behavioural Responses to Thermal Conditions Affect Seasonal Mass Change in a Heat-Sensitive Northern Ungulate. PLOS ONE. 2013 Jun 11;8(6):e65972.

31. Courbin N, Dussault C, Veillette A, Giroux M-A, Côté SD. Coping with strong variations in winter severity: plastic habitat selection of deer at high density. Behav Ecol. 2017 Aug 1;28(4):1037–46.

32. Avgar T, Mosser A, Brown GS, Fryxell JM. Environmental and individual drivers of animal movement patterns across a wide geographical gradient. J Anim Ecol. 2013 Jan 1;82(1):96–106.

33. Kaczensky P, Ganbataar O, Altansukh N, Enkhsaikhan N, Stauffer C, Walzer C. The Danger of Having All Your Eggs in One Basket—Winter Crash of the Re-Introduced Przewalski's Horses in the Mongolian Gobi. PLOS ONE. 2011 Dec 28;6(12):e28057.

34. Luo Z, Liu B, Liu S, Jiang Z, Halbrook RS. Influences of Human and Livestock Density on Winter Habitat Selection of Mongolian Gazelle (Procapra gutturosa). Zoolog Sci. 2014 Jan 1;31(1):20–30.

35. Leclerc M, Vander Wal E, Zedrosser A, Swenson JE, Kindberg J, Pelletier F. Quantifying consistent individual differences in habitat selection. Oecologia. 2016 Mar 1;180(3):697–705.

36. Montgomery RA, Redilla KM, Ortiz-Calo W, Smith T, Keller B, Millspaugh JJ. Evaluating the individuality of animal-habitat relationships. Ecol Evol. 2018;8(22):10893–901.

37. Nandintsetseg D, Bracis C, Olson KA, Böhning-Gaese K, Calabrese JM, Chimeddorj B, et al. Challenges in the conservation of wide-ranging nomadic species. J Appl Ecol. 2019;56(8):1916–26.

38. Olson KA, Fuller TK, Schaller GB, Odonkhuu D, Murray MG. Estimating the population density of Mongolian gazelles Procapra gutturosa by driving long-distance transects. Oryx. 2005 Apr;39(02):164–169.

39. IUCN SSC Antelope Specialist Group. Procapra gutturosa [Internet]. The IUCN Red List of Threatened Species; 2016 [cited 2020 Feb 27]. Available from: https://dx.doi.org/10.2305/IUCN.UK.2016-3.RLTS.T18232A50193126.en

40. Jiang Z, Takatsuki S, Li J, Wang W, Gao Z, Ma J. Seasonal Variations in Foods and Digestion of Mongolian Gazelles in China. J Wildl Manag. 2002 Jan 1;66(1):40–7.

41. Olson KA, Murray MG, Fuller TK. Vegetation Composition and Nutritional Quality of Forage for Gazelles in Eastern Mongolia. Rangel Ecol Manag. 2010 Sep 1;63(5):593–8.

42. Hamel S, Garel M, Festa-Bianchet M, Gaillard J-M, Côté SD. Spring Normalized Difference Vegetation Index (NDVI) predicts annual variation in timing of peak faecal crude protein in mountain ungulates. J Appl Ecol. 2009 Jun 1;46(3):582–9.

43. Pettorelli N, Ryan S, Mueller T, Bunnefeld N, Jędrzejewska B, Lima M, et al. The Normalized Difference Vegetation Index (NDVI): unforeseen successes in animal ecology. Clim Res. 2011 Jan 20;46(1):15–27.

44. Ryan SJ, Cross PC, Winnie J, Hay C, Bowers J, Getz WM. The utility of normalized difference vegetation index for predicting African buffalo forage quality. J Wildl Manag. 2012 Sep 1;76(7):1499–508.

45. Lukacs PM, Mitchell MS, Hebblewhite M, Johnson BK, Johnson H, Kauffman M, et al. Factors influencing elk recruitment across ecotypes in the Western United States. J Wildl Manag. 2018;82(4):698–710.

46. Olson KA, Mueller T, Kerby JT, Bolortsetseg S, Leimgruber P, Nicolson CR, et al. Death by a thousand huts? Effects of household presence on density and distribution of Mongolian gazelles. Conserv Lett. 2011 Aug 1;4(4):304–12.
47. Kawamura K, Akiyama T, Yokota H, Tsutsumi M, Yasuda T, Watanabe O, et al. Comparing MODIS vegetation indices with AVHRR NDVI for monitoring the forage quantity and quality in Inner Mongolia grassland, China. Grassl Sci. 2005 Mar;51(1):33–40.
48. Kawamura, K., Akiyama T, Yokota H, Tsutsumi M, Yasuda T, Watanabe O, et al. Monitoring of forage conditions with MODIS imagery in the Xilingol steppe, Inner Mongolia. Int J Remote Sens. 2005 Apr 1;26(7):1423–36.
49. Garroutte EL, Hansen AJ, Lawrence RL. Using NDVI and EVI to Map Spatiotemporal Variation in the Biomass and Quality of Forage for Migratory Elk in the Greater Yellowstone Ecosystem. Remote Sens. 2016 May;8(5):404.
50. Johnson HE, Gustine DD, Golden TS, Adams LG, Parrett LS, Lenart EA, et al. NDVI exhibits mixed success in predicting spatiotemporal variation in caribou summer forage quality and quantity. Ecosphere. 2018;9(10):e02461.
51. Sawyer H, Kauffman MJ. Stopover ecology of a migratory ungulate. J Anim Ecol. 2011;80(5):1078–87.
52. Manly BB, McDonald L, Thomas D. Resource Selection by Animals: Statistical design and analysis for field studies [Internet]. Springer Netherlands; 1993 [cited 2020 Feb 27]. Available from: https://www.springer.com/gp/book/9780412401404
53. Fortin D, Beyer HL, Boyce MS, Smith DW, Duchesne T, Mao JS. Wolves Influence Elk Movements: Behavior Shapes a Trophic Cascade in Yellowstone National Park. Ecology. 2005 May 1;86(5):1320–30.

54. Ito TY, Lhagvasuren B, Tsunekawa A, Shinoda M, Takatsuki S, Buuveibaatar B, et al. Fragmentation of the Habitat of Wild Ungulates by Anthropogenic Barriers in Mongolia. PLoS ONE. 2013 Feb;8(2):1–9.

55. Fernandez-Gimenez ME. Sustaining the Steppes: A Geographical History of Pastoral Land Use in Mongolia. Geogr Rev. 1999;89(3):315–42.

56. Fernandez-Gimenez ME, Allen-Diaz B. Testing a non-equilibrium model of rangeland vegetation dynamics in Mongolia. J Appl Ecol. 1999 Dec 1;36(6):871–85.

57. Vandandorj S, Munkhjargal E, Boldgiv B, Gantsetseg B. Changes in event number and duration of rain types over Mongolia from 1981 to 2014. Environ Earth Sci. 2017 Jan 1;76(2):70.

58. Fernández-Giménez ME, Batkhishig B, Batbuyan B. Cross-boundary and cross-level dynamics increase vulnerability to severe winter disasters (dzud) in Mongolia. Glob Environ Change. 2012 Oct 1;22(4):836–51.

59. Rao MP, Davi NK, D'Arrigo RD, Skees J, Nachin B, Caroline Leland, et al. Dzuds, droughts, and livestock mortality in Mongolia. Environ Res Lett. 2015;10(7):074012.

60. Berger J, Murray KM, Buuveibaatar B, Dunbar MR, Lkhagvasuren B. Capture of ungulates in Central Asia using drive nets: advantages and pitfalls illustrated by the Endangered Mongolian saiga Saiga tatarica mongolica. Oryx. 2010 Oct;44(04):512–515.

61. Mattiuzzi M, Detsch F. MODIS: Acquisition and Processing of MODIS Products. [software]. 2019. Available from: https://CRAN.R-project.org/package=MODIS

62. R Core Team. R: A language and environment for statistical computing. [software]. Vienna, Austria: R Foundation for Statistical Computing; 2020. Available from: https://www.R-project.org/

63. Calenge C. The package “adehabitat” for the R software: A tool for the analysis of space and habitat use by animals. Ecol Model. 2006 Aug;197(3–4):516–9.

64. Bivand R, Rundel C, Pebesma E, Stuetz R, Hufthammer KO, Giraudoux P, et al. rgeos: Interface to Geometry Engine - Open Source ('GEOS’) [software]. 2020 [cited 2020 Aug 13]. Available from: https://CRAN.R-project.org/package=rgeos

65. Hijmans RJ, Phillips S, Elith JL and J. dismo: Species Distribution Modeling [software]. 2017 [cited 2020 Aug 13]. Available from: https://CRAN.R-project.org/package=dismo

66. Bivand R, Pebesma E, Gomez-Rubio V. Applied spatial data analysis with R [software]. second. Springer NY; 2013. Available from: https://asdar-book.org/

67. Pebesma E, Bivand R, Rowlingson B, Gomez-Rubio V, Hijmans R, Sumner M, et al. sp: Classes and Methods for Spatial Data [software]. 2020 [cited 2020 Aug 13]. Available from: https://CRAN.R-project.org/package=sp

68. Barbet-Massin M, Jiguet F, Albert CH, Thuiller W. Selecting pseudo-absences for species distribution models: how, where and how many? Methods Ecol Evol. 2012;3(2):327–38.

69. Bates D, Mächler M, Bolker B, Walker S. Fitting Linear Mixed-Effects Models Using lme4. J Stat Softw. 2015 Oct 7;67(1):1–48.

70. Fithian W, Hastie T. Finite-Sample Equivalence in Statistical Models for Presence-Only Data. Ann Appl Stat. 2013 Dec 1;7(4):1917–39.

71. Johnson DH. The Comparison of Usage and Availability Measurements for Evaluating Resource Preference. Ecology. 1980;61(1):65–71.

72. Thurfjell H, Ciuti S, Boyce MS. Applications of step-selection functions in ecology and conservation. Mov Ecol. 2014 Feb 7;2(1):4.

73. Muff S, Signer J, Fieberg J. Accounting for individual-specific variation in habitat-selection studies: Efficient estimation of mixed-effects models using Bayesian or frequentist computation. J Anim Ecol. 2020;89(1):80–92.

74. Therneau T. A Package for Survival Analysis in R [software]. 2020. Available from: https://CRAN.R-project.org/package=survival

75. Therneau TM, Grambsch PM. Modeling survival data: extending the cox model. New York: Springer-Verlag; 2000

76. Avgar T, Lele SR, Keim JL, Boyce MS. Relative Selection Strength: Quantifying effect size in habitat- and step-selection inference. Ecol Evol. 2017 Jul 1;7(14):5322–30.

77. Dye DG, Tucker CJ. Seasonality and trends of snow-cover, vegetation index, and temperature in northern Eurasia. Geophys Res Lett. 2003 Apr 11;30(7):58.

78. Pettorelli N, Vik JO, Mysterud A, Gaillard J-M, Tucker CJ, Stenseth NChr. Using the satellite-derived NDVI to assess ecological responses to environmental change. Trends Ecol Evol. 2005 Sep;20(9):503–10.

79. Nanzad L, Zhang J, Tuvdendorj B, Nabil M, Zhang S, Bai Y. NDVI anomaly for drought monitoring and its correlation with climate factors over Mongolia from 2000 to 2016. J Arid Environ. 2019 May;164:69–77.

80. Squires JR, DeCesare NJ, Olson LE, Kolbe JA, Hebblewhite M, Parks SA. Combining resource selection and movement behavior to predict corridors for Canada lynx at their southern range periphery. Biol Conserv. 2013 Jan 1;157:187–95.

81. Reinking AK, Smith KT, Mong TW, Read MJ, Beck JL. Across scales, pronghorn select sagebrush, avoid fences, and show negative responses to anthropogenic features in winter. Ecosphere. 2019 May 1;10(5):e02722.

82. Talbot LM, Talbot MH. The Wildebeest in Western Masailand, East Africa. Wildl Monogr. 1963;(12):3–88.

83. Martínez-García R, Calabrese JM, Mueller T, Olson KA, López C. Optimizing the Search for Resources by Sharing Information: Mongolian Gazelles as a Case Study. Phys Rev Lett. 2013 Jun 14;110(24):248106.

84. Kareiva P, Odell G. Swarms of Predators Exhibit “Preytaxis” if Individual Predators Use Area-Restricted Search. Am Nat. 1987 Aug 1;130(2):233–70.

85. Morales JM, Haydon DT, Frair J, Holsinger KE, Fryxell JM. Extracting more out of relocation data: building movement models as mixtures of random walks. Ecology. 2004 Sep 1;85(9):2436–45.

86. Avgar T, Potts JR, Lewis MA, Boyce MS. Integrated step selection analysis: bridging the gap between resource selection and animal movement. Methods Ecol Evol. 2016 May 1;7(5):619–30.

87. Patterson TA, Thomas L, Wilcox C, Ovaskainen O, Matthiopoulos J. State–space models of individual animal movement. Trends Ecol Evol. 2008 Feb;23(2):87–94.

88. Gurarie E, Andrews RD, Laidre KL. A novel method for identifying behavioural changes in animal movement data. Ecol Lett. 2009;12(5):395–408.

89. Jones PF, Jakes AF, Telander AC, Sawyer H, Martin BH, Hebblewhite M. Fences reduce habitat for a partially migratory ungulate in the Northern Sagebrush Steppe. Ecosphere. 2019;10(7):e02782.

90. Muntifering JR, Ditmer MA, Stapleton S, Naidoo R, Harris TH. Hartmann’s mountain zebra resource selection and movement behavior within a large unprotected landscape in northwest Namibia. Endanger Species Res. 2019 Mar 28;38:159–70.

91. Tsalyuk M, Kilian W, Reineking B, Getz WM. Temporal variation in resource selection of African elephants follows long-term variability in resource availability. Ecol Monogr. 2019 May 1;89(2):e01348.
92. Lesmerises R, St-Laurent M-H. Not accounting for interindividual variability can mask habitat selection patterns: a case study on black bears. Oecologia. 2017 Nov 1;185(3):415–25.
93. Mumma MA, Gillingham MP, Johnson CJ, Parker KL. Understanding predation risk and individual variation in risk avoidance for threatened boreal caribou. Ecol Evol. 2017;7(23):10266–77.
94. Zeller KA, Wattles DW, Conlee L, DeStefano S. Black bears alter movements in response to anthropogenic features with time of day and season. Mov Ecol. 2019 Jul 11;7(1):19.
95. Schoener TW. Theory of Feeding Strategies. Annu Rev Ecol Syst. 1971;2(1):369–404.
96. Bergman CM, Fryxell JM, Gates CC, Fortin D. Ungulate Foraging Strategies: Energy Maximizing or Time Minimizing? J Anim Ecol. 2001;70(2):289–300.

Supporting Information Captions

S1 Appendix. Effect of the number of random points on step-selection function coefficients.

S2 Appendix. Analysis examining differences in NDVI between areas selected by and available to Mongolian gazelles.

S1 Fig. The effect of the number of pseudo-absence points on model coefficients of the RSFs. Models were run with an equal number of presence and pseudo-absence points, double the number of pseudo-absence points as presence points, and 5, 10, and 20 times the number of pseudo-absence points as presence points. This was done for all models. Here we show the results for the RSF for each season which had the most support based on AIC. A) For the growing

season this was a quadratic model (NDVI + NDVI^2) with no random effects. B) For winter, this as a quadratic model (snow cover + snow cover^2) with random slopes for individual gazelles. Beta coefficients for the linear and quadratic parameter are shown. Differences between 10 and 20 times the number of pseudo-absence points are small so we present results of models run with 20 times more pseudo-absence points than presence points.

S2 Fig. Selection for NDVI at the 1 day step scale shown by individual Mongolian gazelles in a given year during the growing season (May 9 – Aug. 29). Selection is quantified as relative selection strength (RSS). Curves were plotted based on the results of the individual-year SSFs and show the best fit model for a given individual-year combination. Only those individuals who showed significant selection at the 1 day step-scale are shown. The vertical line indicates the mean NDVI (as described in the methods), which was used as the reference point to create the curves. Dots indicate the most preferred NDVI values compared to the mean NDVI. The curve is only plotted for the range of NDVI available to the individual gazelle as defined in the methods.

S3 Fig. Differences among Mongolian gazelles in selection for NDVI during the growing season at the 1 day step scale based on step-selection functions (SSFs) run for each individual-year combination. The majority of Mongolian gazelles did not show selection at the 1 day step scale. Blue and yellow sections of the bar plots show the percent of individuals selecting for NDVI during the growing season of a given year or for snow cover during winter of a given year, grey shows the percent of individuals that did not show selection. For those individuals showing selection, dark blue indicates selection for the highest NDVI (or snow cover) available, light blue indicates selection for the lowest NDVI (or snow cover) available, and yellow selection for intermediate values. Above each bar are the number of individuals which had sufficient data (minimum of 20 steps) to run step-selection functions on an individual-year basis.

S4 Fig. Selection for NDVI at the 5 day step scale shown by individual Mongolian gazelles during the growing season (May 9 – Aug. 29). Selection is quantified as relative selection strength (RSS). Curves were plotted based on the results of the individual SSFs and show the best fit model for an individual. Only those individuals who showed significant selection at the 5 day step-scale are shown. The vertical line indicates the mean NDVI (as described in the methods), which was used as the reference point to create the curves. Dots indicate the most preferred NDVI values compared to the mean NDVI. The curve is only plotted for the range of NDVI available to the individual gazelle as defined in the methods.

S5 Fig. Selection for NDVI at the 10 day step scale shown by individual Mongolian gazelles during the growing season (May 9 – Aug. 29). Selection is quantified as relative selection strength (RSS). Curves were plotted based on the results of the individual SSFs and show the best fit model for an individual. Only those individuals who showed significant selection at the 10 day step-scale are shown. The vertical line indicates the mean NDVI (as described in the methods), which was used as the reference point to create the curves. Dots indicate the most preferred NDVI values compared to the mean NDVI. The curve is only plotted for the range of NDVI available to the individual gazelle as defined in the methods.

S6 Fig. Selection for snow cover at the 1 day step scale shown by individual Mongolian gazelles in a given year during the winter (Aug. 30 – May 8). This figure displays all individuals showing significant selection. Selection was quantified as relative selection strength (RSS). Curves were plotted based on the results of the individual SSFs and show the best fit model for an individual. The vertical line indicates mean snow cover (as described in the methods), which was used as the reference point to create the curves. Dots indicate the most preferred snow cover value compared to mean snow cover. The curve is only plotted for the range of snow cover available to the individual gazelle as defined in the methods.

S7 Fig. Selection for snow cover at the 5 day step scale shown by individual Mongolian gazelles in a given year during the winter (Aug. 30 – May 8). Selection was quantified as relative selection strength (RSS). Curves were plotted based on the results of the individual SSFs and show the best fit model for an individual. Only those individuals who showed significant selection at the 5 day step-scale are shown. The vertical line indicates mean snow cover (as described in the methods), which was used as the reference point to create the curves. Dots indicate the most preferred snow cover value compared to mean snow cover. The curve is only plotted for the range of snow cover available to the individual gazelle as defined in the methods. The figure on the right is zoomed in to better show the shape of selection for the majority of individuals.

S8 Fig. Selection for snow cover at the 10 day step scale shown by individual Mongolian gazelles in a given year during the winter (Aug. 30 – May 8). Selection was quantified as relative selection strength (RSS). Curves were plotted based on the results of the individual SSFs and show the best fit model for an individual. Only those individuals who showed significant selection at the 10 day step-scale are shown. The vertical line indicates mean snow cover (as described in the methods), which was used as the reference point to create the curves. Dots indicate the most preferred snow cover value compared to mean snow cover. The curve is only plotted for the range of snow cover available to the individual gazelle as defined in the methods. The figure on the right is zoomed in to better show the shape of selection for the majority of individuals.

S1 Table. Beta coefficients for the best fit RSF model describing selection by Mongolian gazelles for NDVI during the growing season (May 9 – Aug. 29). The quadratic model was based on 3379 daily locations from 25 individuals over 3 years.

S2 Table. Beta coefficients for the best fit RSF model describing selection by Mongolian gazelles for snow cover during winter (Aug. 30 – May 8). The quadratic model was based on 9791 daily locations from 33 individuals over four winters. The table presents the beta coefficients, standard error, and p-values for the overall model.

S3 Table. Variance (with standard deviation) explained by the random effects in the RSF model describing selection by Mongolian gazelles for snow cover during winter (Aug. 30 – May 8).

S4 Table. Number of steps for the growing season SSFs for each individual gazelle over the three step-length scales tests.

S5 Table. Number of steps for the growing season SSFs run by individual/year at the 1 day step-length scale.

S6 Table. Number of steps for the winter SSFs run by individual/year over the three step-length scales tests.

S7 Table. AIC values for the different growing season (May 9 – Aug. 29) SSF models examining how Mongolian gazelles select for NDVI. Models represent different hypotheses on how Mongolian gazelles select for NDVI (linear or quadratic). A linear model has just NDVI as an explanatory variable. A quadratic model has NDVI + $NDVI^2$ as explanatory variables. Models were run for each individual with 1, 5, and 10 day step-lengths. For the 1 day step length we were also able to run models by individual-year. Results are shown only for those individuals where the top-model had a significant p-value ($\alpha < 0.05$). ΔAIC is the difference between a model and the top model.

S8 Table. AIC values for the different winter (Aug. 30 – May 8) SSF models examining how Mongolian gazelles select for snow cover. Models represent different hypotheses on how Mongolian gazelles select for snow cover (linear or quadratic). Models were run for each

individual-year with 1, 5, and 10 day step-lengths. Results are shown only for those individual-years where the top-model had a significant p-value ($\alpha < 0.05$). ΔAIC is the difference between a model and the top model.

Figure 1

Click here to access/download;Figure;Fig1.tiff

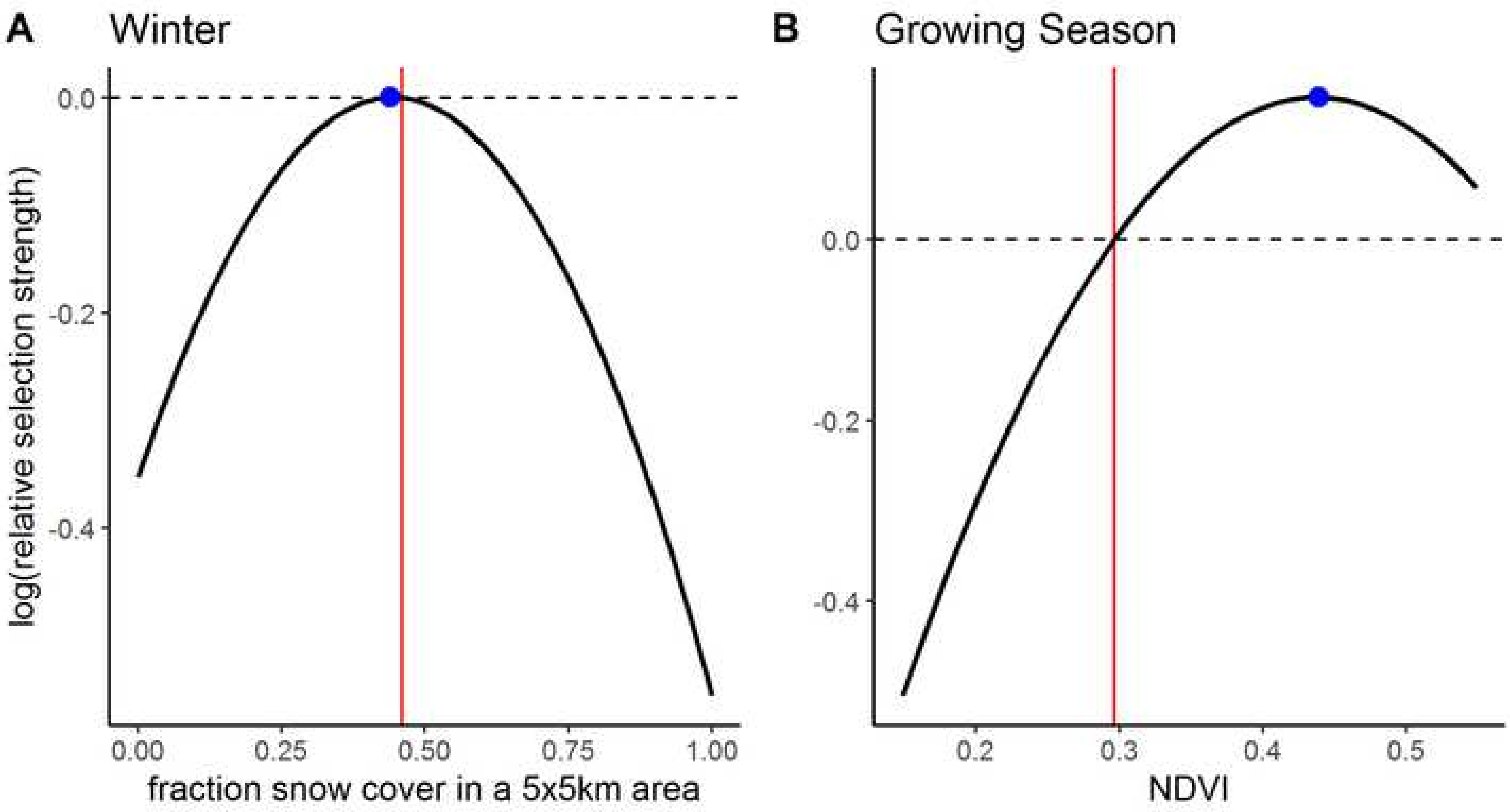

Figure 2

Click here to access/download;Figure;Fig2.tiff

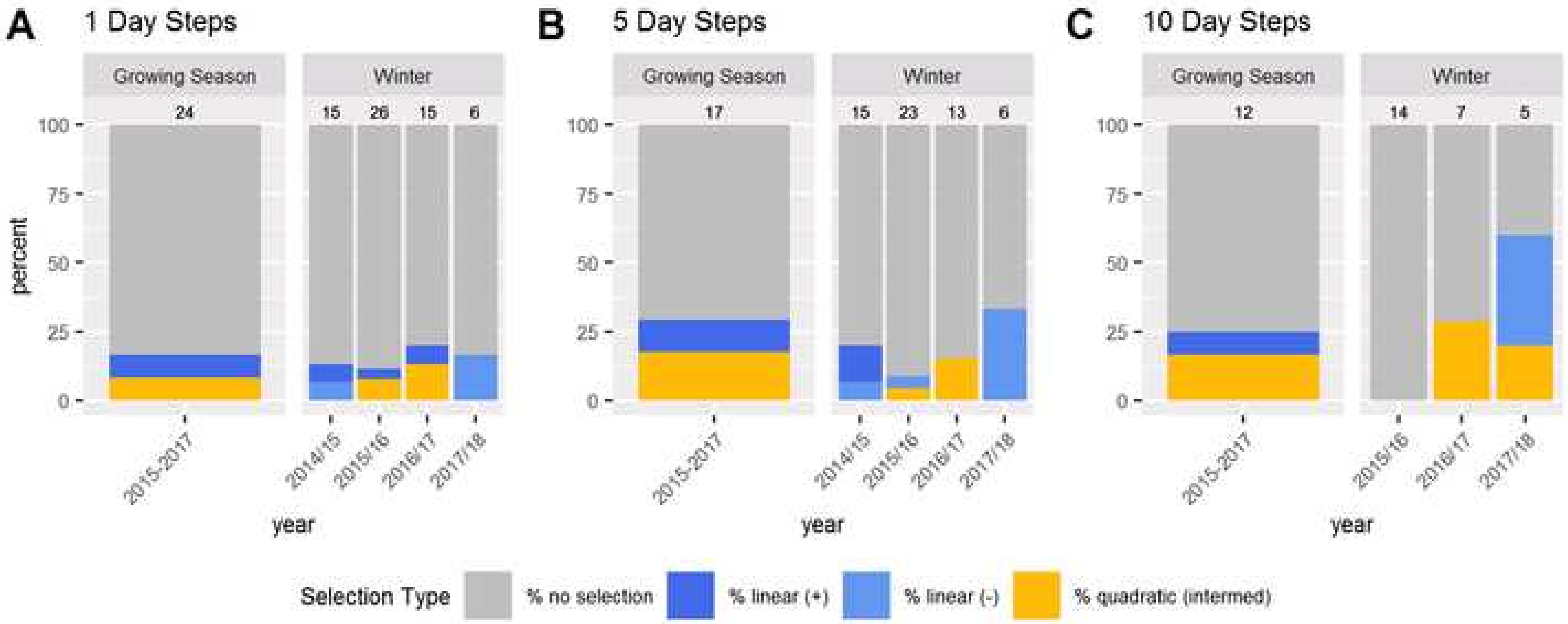

Figure 3

Click here to access/download;Figure;Fig3.tiff

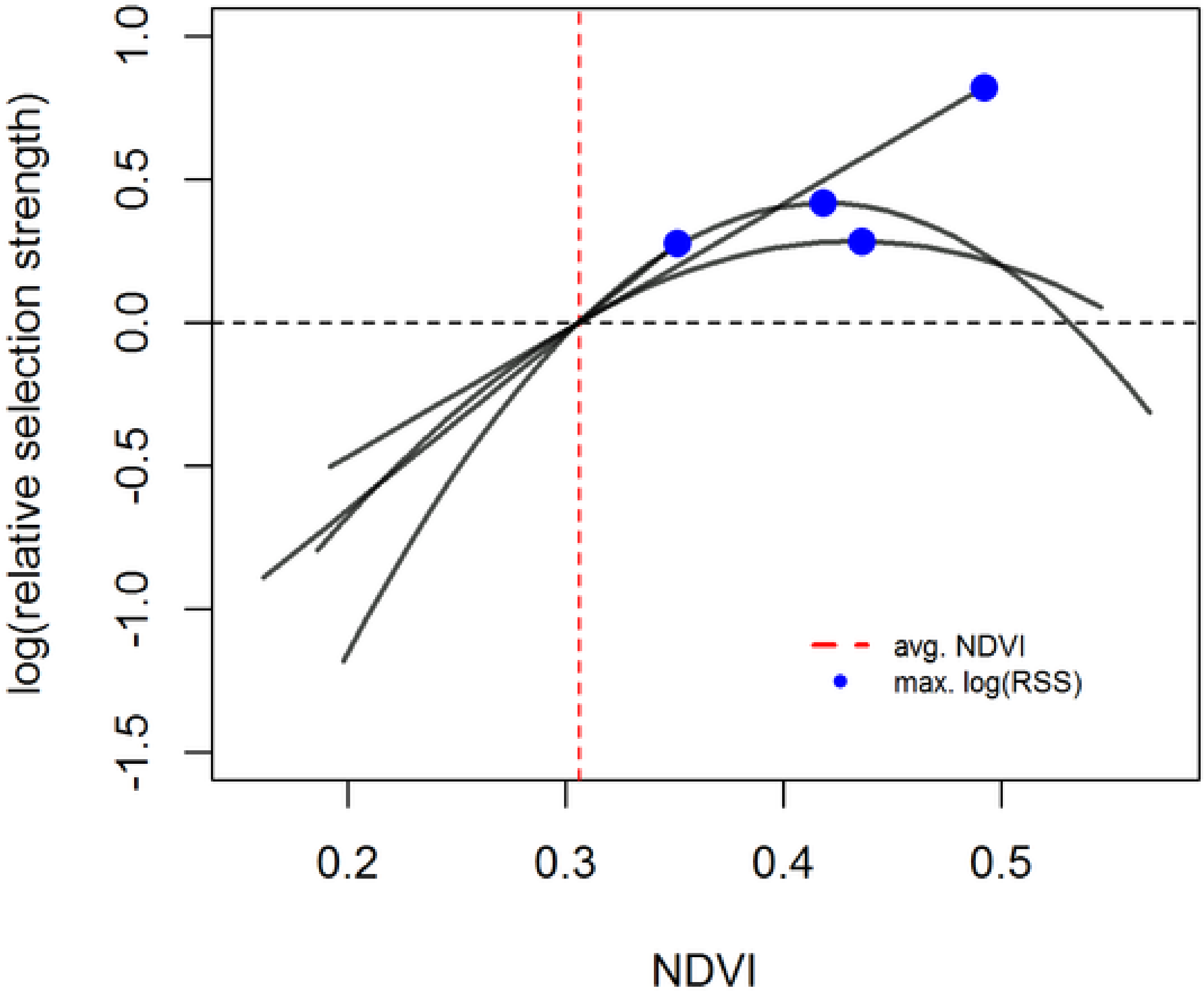

Figure 4

Click here to access/download;Figure;Fig4.tiff

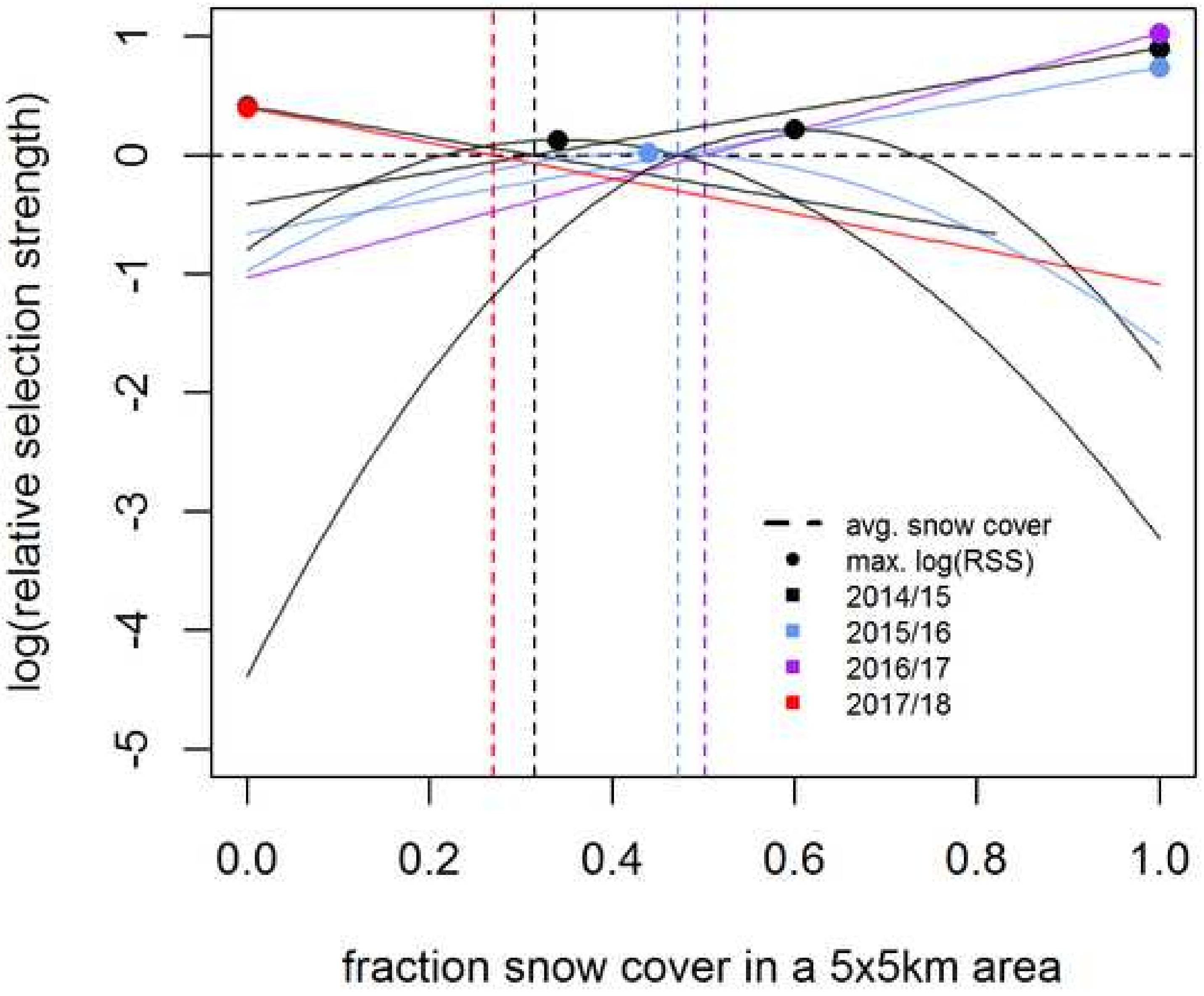

Figure 5

Click here to access/download;Figure;Fig5.tiff

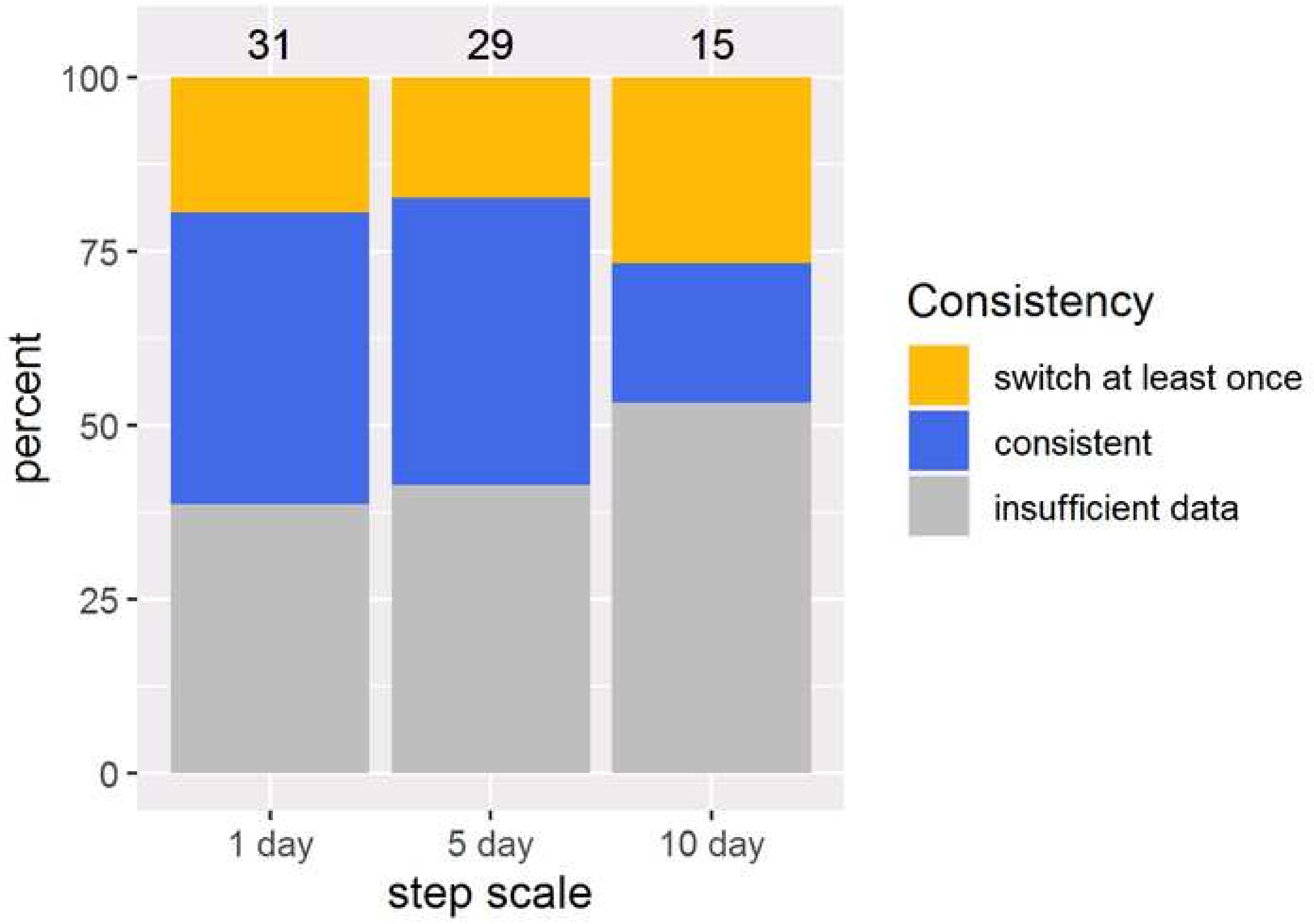

Supporting Information Appendix

Click here to access/download
Supporting Information
S1_Appendix.pdf

Supporting Information Appendix

Click here to access/download
Supporting Information
S2_Appendix.pdf

Supporting Information Figure

Click here to access/download
Supporting Information
S1_Fig.tiff

Supporting Information Figure

Click here to access/download
Supporting Information
S2_Fig.tiff

Supporting Information Figure

Click here to access/download
Supporting Information
S3_Fig.tiff

Supporting Information Figure

Click here to access/download
Supporting Information
S4_Fig.tiff

Supporting Information Figure

Click here to access/download
Supporting Information
S5_Fig.tiff

Supporting Information Figure

Click here to access/download
Supporting Information
S6_Fig.tiff

Supporting Information Figure

Click here to access/download
Supporting Information
S7_Fig.tiff

Supporting Information Figure

Click here to access/download
Supporting Information
S8_Fig.tiff

Click here to access/download
Supporting Information
S1_Table.xlsx

Supporting Information Table

Click here to access/download
Supporting Information
S2_Table.xlsx

Supporting Information Table

Click here to access/download
Supporting Information
S3_Table.xlsx

Supporting Information Table

Click here to access/download
Supporting Information
S4_Table.xlsx

Supporting Information Table

Click here to access/download
Supporting Information
S6_Table.xlsx

Supporting Information Table

Click here to access/download
Supporting Information
S7_Table.xlsx

Supporting Information Table

Click here to access/download
Supporting Information
S8_Table.xlsx

Supporting Information Table

Click here to access/download
Supporting Information
S5_Table.xlsx
